# Abnormal B cell glycosylation in autoimmunity: A new potential treatment strategy

**DOI:** 10.3389/fimmu.2022.975963

**Published:** 2022-08-25

**Authors:** Marie Morel, Pierre Pochard, Wiam Echchih, Maryvonne Dueymes, Cristina Bagacean, Sandrine Jousse-Joulin, Valérie Devauchelle-Pensec, Divi Cornec, Christophe Jamin, Jacques-Olivier Pers, Anne Bordron

**Affiliations:** ^1^ LBAI, UMR1227, Univ Brest, Inserm, Brest, France; ^2^ Laboratoire d'Immunologie et d'Immunothérapie, CHU de Brest, Brest, France

**Keywords:** glycosylation, fucosylation, sialylation, N-glycosylation, O-glycosylation, autoimmune diseases, primary Sjogren’s syndrome, systemic lupus erythematosus

## Abstract

Systemic lupus erythematosus (SLE) and primary Sjögren’s syndrome (pSS) are two autoimmune diseases characterised by the production of pathogenic autoreactive antibodies. Their aetiology is poorly understood. Nevertheless, they have been shown to involve several factors, such as infections and epigenetic mechanisms. They also likely involve a physiological process known as glycosylation. Both SLE T cell markers and pSS-associated autoantibodies exhibit abnormal glycosylation. Such dysregulation suggests that defective glycosylation may also occur in B cells, thereby modifying their behaviour and reactivity. This study aimed to investigate B cell subset glycosylation in SLE, pSS and healthy donors and to extend the glycan profile to serum proteins and immunoglobulins. We used optimised lectin-based tests to demonstrate specific glycosylation profiles on B cell subsets that were specifically altered in both diseases. Compared to the healthy donor B cells, the SLE B cells exhibited hypofucosylation, whereas only the pSS B cells exhibited hyposialylation. Additionally, the SLE B lymphocytes had more galactose linked to N-acetylglucosamine or N-acetylgalactosamine (Gal-GlcNAc/Gal-GalNAc) residues on their cell surface markers. Interestingly, some similar alterations were observed in serum proteins, including immunoglobulins. These findings indicate that any perturbation of the natural glycosylation process in B cells could result in the development of pathogenic autoantibodies. The B cell glycoprofile can be established as a preferred biomarker for characterising pathologies and adapted therapeutics can be used for patients if there is a correlation between the extent of these alterations and the severity of the autoimmune diseases.

## 1 Introduction

Glycobiology is the study of the structure, biosynthesis and biology of glycans. Glycosylation is involved in numerous physiological processes, including cell proliferation, differentiation and apoptosis. It also participates in the intracellular trafficking of several glycoproteins and directs them to their intended destination (1). Glycoconjugates are formed when sugars are added to proteins and lipids, and this phenomenon consists of a succession of enzymatic reactions that mainly occur in the endoplasmic reticulum (ER) and Golgi apparatus. These reactions are specifically performed by glycosyltransferases, which catalyse the transfer of substrates to well-defined sites, and glycosidases, which hydrolyse glycosidic bonds (2).

Saccharides bind to proteins through a wide variety of ligations mediated by N-glycan and O-glycan bonds. The N-glycan bond is initiated in the ER and occurs between the N-acetylglucosamine (GlcNAc) and the NH2 residue of asparagine (Asn). This bound defines the binding site for various complex oligosaccharides on proteins (3, 4). The heterogeneity of N-glycan synthesis in eukaryotes depends on the cell developmental state and the availability of glycosyltransferases, glycosidases and glycans. O-glycan bonds consist of hydroxyl groups of various amino acids (including serine, threonine and tyrosine) and some monosaccharides such as N-acetylgalactosamine (GalNAc), GlcNAc or galactose (Gal). They mainly occur in the Golgi apparatus and use glycosyl nucleotides (e.g. cytidine 5’- monophosphate [CMP-SIA]) as a donor substrate (5). N and O-glycosylation can occur on the same glycoprotein.

**Figure 1:**
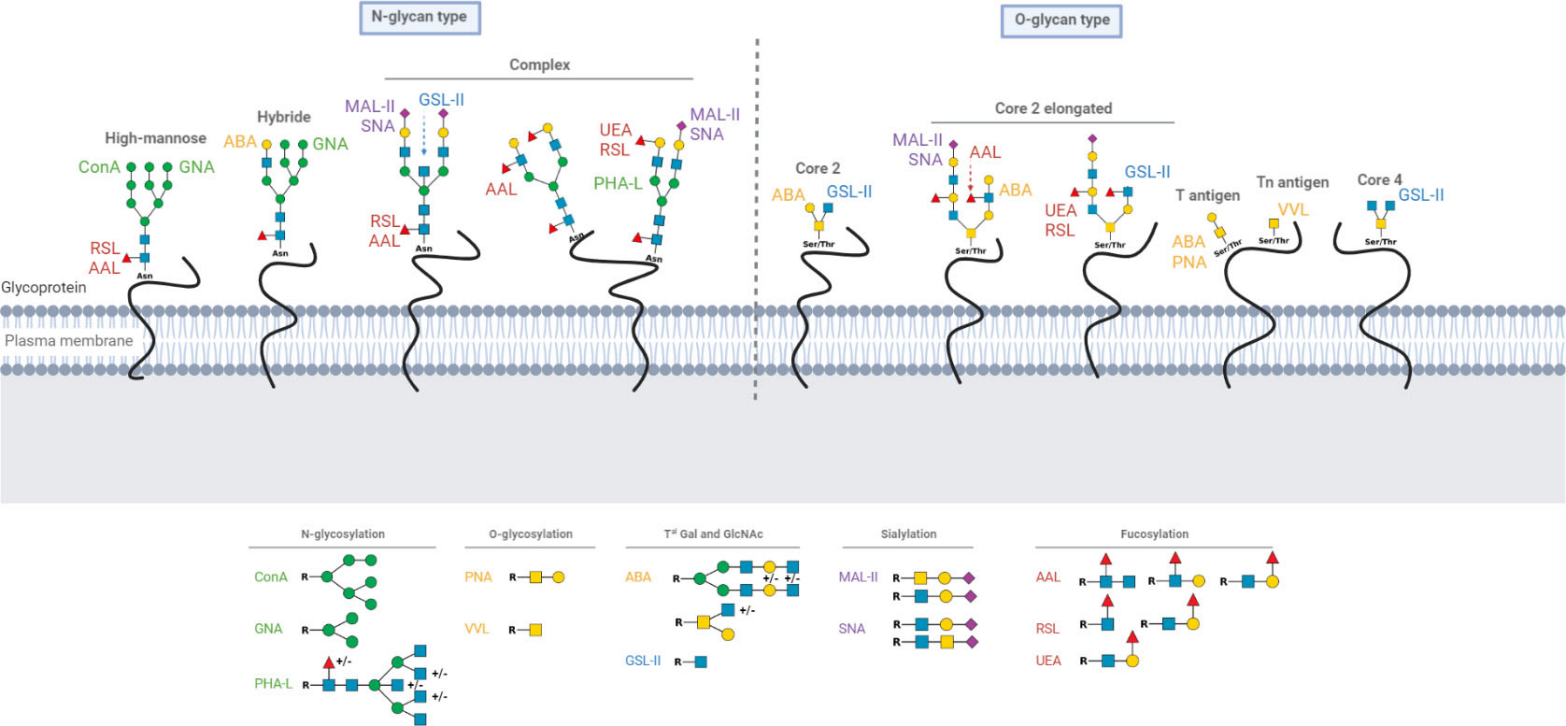
Schematic representation of N and O-glycoproteins on cell surface and glycan motives recognized by lectins.The different known forms of N and O-glycoproteins on cell surface are presented as well as glycan motives recognized by lectins. Green circle: Mannose; yellow circle: Galactose; yellow square: N-acetylgalactosamine; blue square: N-acetylglucosamine; purple diamond; Sialic acid and red triangle; Fucose. Asn, Asparagine; Thr, Threonine; Ser, Serine.

Many proteins are modified by N-glycosylation on Asn, which can bind various supplementary monosaccharides, as well as galactosylation, GlcNAclyation, sialylation and fucosylation, all of which determine whether the final structure would be a high-mannose N-glycan, a hybrid N-glycan or a complex N-glycan. However, the glycopeptide O-glycan chains are modified by distinct glycosyltransferases that can elongate the existing structure with Gal and GlcNAc to form different core molecules as well as expand cores with sialic acid and fucose (6) (**Figure 1**).

Changes in glycosylation have been stated to cause the development of cancers. The modulation of the sialylation of different cell surface receptors promotes survival, proliferation, metastasis development and resistance to drugs and chemotherapy (7–10). In addition to sialylation, aberrant fucosylation and a decrease in GlcNAcylation appear to play a role in solid cancer evolution (11, 12). This aberrant glycosylation has been proposed as a biomarker to predict malignant emergence and cancer progression (13, 14).

Based on these findings, we hypothesised that changes in protein glycosylation could be involved in the development of autoimmune diseases (AIDs), such as primary Sjögren’s syndrome (pSS) and systemic lupus erythematosus (SLE). These two chronic systemic diseases, which mainly affect women, are characterised by the production of pathogenic autoantibodies (15, 16). Their aetiology is quite complex, multi-factorial and still unknown. It involves genetic mutations, epigenetic modifications and viral infections (17).

The chronic AID known as pSS is characterised by the development of clinical symptoms on mucosal surfaces, such as the mouth and eyes. It is also called ‘sicca syndrome’ because of the progressive destruction of exocrine glands and the emergence of excessive dryness (18). The severity of the disease influences the composition of the salivary gland infiltrates. B and T cells are mainly present in mild lesions, whereas antigen-presenting cells and natural killer cells are present in severe lesions (19). The abnormal abundance of follicular helper T cells, follicular regulatory T cells and plasma cells has also been described (20). B cells play a key role in pSS pathogenesis because they secrete anti-Ro/SSA and La/SSB autoantibodies (15). Glycosylation contributes to the selection of autoreactive B cells since somatic mutations result in the appearance of new N-glycosylation sites and antibodies that can bind autoantigens. The secretion of the N-glycosylation-mutated fragment antigen-binding region of IgG is associated with B cell activation in salivary glands, confirming the importance of the glycoprofile in the development of pSS (21).

SLE is another chronic AID characterised by the production of autoantibodies with various clinical manifestations. The main pathological features in SLE patients are inflammation, immune complex deposits and organs damages, which can lead to nephropathy (22). The severity of SLE has been linked to abnormal T cell subset distribution, such as defects in regulatory T cells, and altered functions (23, 24). B lymphocytes are also actively involved in the development of SLE through different aspects, including the production of pathogenic autoantibodies (25). SLE B cells are also believed to cause disrupted regulation functions. Compared to healthy donors (HDs), SLE patients have B cells that are less efficient in regulating the proliferation of T cells and reducing the production of T helper 1 (Th1) cytokines such as tumour necrosis factor (TNF) alpha and interferon (IFN) gamma when they are co-cultured with autologous T cells (26–28). Moreover, antibodies from SLE patients show glycoprofile abnormalities. Asialylation of IgA results in poor clearance and excessive immune complex accumulation in the kidney, causing chronic inflammation and organ lesions, including nephritis (29, 30).

Based on these findings, we designed a method to evaluate the N-glycosylation and O-glycosylation status of B cells, their subsets and the produced IgA1, IgA2 and IgG immunoglobulins from pSS and SLE patients in comparison to HDs using flow cytometry and ELISA-derived approaches that combine lectins.

## 2 Material and methods

### 2.1 Design of control and patient groups

Tonsillar mononuclear cells (TMCs) were isolated from 20 HDs who underwent a routine tonsillectomy at either CHU Morvan or Clinique Pasteur (Brest, France). Peripheral blood mononuclear cells (PBMCs) were isolated from the blood samples of 30 HDs obtained at the Etablissement Français du Sang (Brest, France), 10 pSS patients fulfilling the American College of Rheumatology (ACR)/European Alliance of Association for Rheumatology (EULAR) 2016 criteria and 17 SLE patients fulfilling the ACR/EULAR 2019 criteria. The blood samples from the pSS and SLE patients were collected from the ‘CRB Santé de Brest’ (BB-0033-00037) and ‘Centre de référence des maladies auto-immunes rares’ in Brest, France. All patients agreed to participate in the research protocol by signing a consent form.

A cross-matched selection of 13 HDs (12 females and 1 randomly selected male) was performed to make a reliable comparison with autoimmune patients.

For the serum analysis, 10 HD, 10 pSS and 10 SLE sera were obtained from the European PRECISESADS study (Innovative Medicine Initiative Joint Undertaking under the grant agreement number 115565).

All characteristics are described in **Table 1**.

**Table 1:** Demographics and disease activity parameters.

Items	Tonsil HD (n=20)	Blood HD (n=30)	Blood pSS (n=10)	Blood SLE (n=17)	Serum HD (n=10)	Serum pSS (n=10)	Serum SLE (n=10)
Female	MD	12	9	16	9	10	9
Male	MD	18	1	1	1	0	1
Age (mean)	<10	39,6	52,3	44,8	36	67,2	42,8
Disease activity score	N/A	N/A	ESSDAI score [4;6] n=6 [7;8] n=4	SLEDAI score [11;16] n=9 [17;34] n=8	N/A	ESSDAI score MD n= 3 [0;4] n=4 [5;10] n=3	SLEDAI score MD n= 2 [0;4] n=6 [5;10] n=2

HD, Healthy Donors; pSS, primary Sjögren’s syndrome; SLE, Systemic lupus erythematosus; ESSDAI, Eular Sjögren Syndrome Disease Activity Index; SLEDAI, Systemic Lupus Erythematosus Disease Activity Index; N/A, not applicable; MD, missing data.

### 2.2 Isolation of mononuclear cells

A 5 mL syringe plunger was used to mince and mash tonsils into a 40 µm nylon mesh in phosphate buffer saline (PBS) solution (Eurobio scientific, Les Ulis, France). The cell suspension was layered on density gradient Pancoll (PAN-Biotech GmbH, Aidenbach, Germany) and uninterruptedly centrifuged at 750 g and room temperature (RT) for 20 min. The isolated cells were washed twice with PBS and counted using a Malassez haemocytometer.

Platelets were removed from the blood samples by low-speed centrifugation (300 g, RT and 15 min). After discarding the supernatant, the blood samples were diluted in half and layered on density gradient Pancoll. After centrifugation at 750 g (RT, 20 min), the isolated cells were washed twice in PBS and counted using the Malassez haemocytometer.

### 2.3 Cell culture

Burkitt lymphoma Ramos B cell lines were grown in an RPMI-1640 solution (Eurobio scientific, Les Ulis, France) containing 10% fetal bovine serum (Eurobio scientific, Les Ulis, France), 50 U/mL penicillin (Panpharma, La Selle-en-Luitré, France), 50 mg/mL streptomycin (Sigma-Aldrich, St. Louis, MI, USA) and 2 mM L-glutamine (Gibco, Thermo Scientific, Waltham, MA, USA) at 37°C in a humidified 5% CO_2_ atmosphere.

### 2.4 Lectin inhibitory binding tests

Several vegetal lectins were described and used to recognise specific saccharides and their complexity. Agaricus bisporus agglutinin (ABA), Ralstonia solanacearum lectin (RSL) and Sambucus nigra agglutinin (SNA) were purchased from GLYcoDiag (Orléans, France). Aleuria aurantia lectin (AAL), Canavalia ensiformis agglutinin (ConA), Galanthus nivalis agglutinin (GNA), Griffonia (Bandeiraea) simplicifolia lectin-II (GSL-II), Maackia amurensis lectin-II (MAL-II), Phaseolus vulgaris leucoagglutinin (PHA-L), Arachis (peanut) hypogaea agglutinin (PNA), Ulex europaeus agglutinin (UEA) and Vicia villosa lectin (VVL) were obtained from Vector Laboratories, Inc. (Burlingame, CA, USA; **Table 2**). All lectins were biotinylated. Monosaccharides were used to validate the specificity of the lectin binding. Lectins were pre-incubated with a PBS solution containing monosaccharides at 4°C for 15 min (**Table 2**) before being used to stain the Ramos cell line for 15 min at 4°C. The cells were washed twice with PBS and subsequently incubated at 4°C for 15 min using 5 µg/mL streptavidine-fluorescein isothiocyanate (FITC) (Biolegend, San Diego, CA, USA). The cells were washed with PBS and analysed using a Beckman Coulter Cytoflex S flow cytometer (Beckman Coulter, Brea, CA, USA).

**Table 2:** Lectin specificities.

Complete name	Abbreviation	Motif binding	Reference	Concentration of monosaccharide and neuraminidase used for inhibitory tests
*Aleuria aurantia* lectin	AAL	GlcNAc(β1-4)[Fuc(α1-6])GlcNAc Fuc(α1-3)Gal(β1-4)GlcNAc Gal(β1-4)[Fuc(α1-3)]GlcNAc Gal(β1-3)[Fuc(α1-4)]GlcNAc	(31–33)	200mM of L-Fucose^‡^
*Agaricus bisporus* agglutinin	ABA	GlcNAc(β) Gal(β1-3)GalNAc-Ser/Thr Gal(β1-4)GlcNAc Gal(β1-3)[GlcNAc(β1-6)]GalNAc-Ser/Thr	(33–35)	83mM of N-Acetylgalactosamine^*^
*Canavalia ensiformis* agglutinin	ConA	Man(α)	(33, 36)	360mM of α-Methyl-Mannoside^*^
*Galanthus nivalis* agglutinin	GNA	Man(α1-3) Man(α1-6)	(33, 36)	360mM of α-Methyl-Mannoside^*^
*Griffonia (Bandeiraea) simplicifolia* lectin-II	GSL-II	GlcNAc(α/β)	(33, 37)	200mM of N-Acetylglucosamine^*^
*Maackia amurensis* lectin-II	MAL-II	Neu5Ac(α2-3)Gal(β1-4)GlcNAc Neu5Ac(α2-3)Gal(β1-3)GalNAc	(33, 38, 39)	0.05U/mL of Neuraminidase^‡^
*Phaseolus vulgaris* leucoagglutinin	PHA-L	GlcNAc(β1-6)Man(α1-6)	(33, 40)	720mM of D-Galactose^‡^
*Arachis (Peanut) hypogaea* agglutinin	PNA	Gal(β1-3)GalNAc-Ser/Thr (*Thomsen-* *Friedenreich (T) antigen*)	(33, 41)	200mM of D-Galactose^‡^
*Ralstonia solanacearum* lectin	RSL	Fuc(α1-2)Gal Fuc(α1-6)GlcNAc	(42)	200mM of L-Fucose^‡^
*Sambucus nigra* agglutinin	SNA	Neu5Ac(α2-6)Gal(β1-4)GlcNAc Neu5Ac(α2-6)GalNAc(β1-4)GlcNAc	(33, 38, 39)	0.05U/mL of Neuraminidase^‡^
*Ulex europaeus* agglutinin	UEA	[Fuc(α1-2)]Gal(β1-4)GlcNAc	(33, 41)	200mM of L-Fucose^‡^
*Vicia villosa* lectin	VVL	GalNAc(α/β) (*Tn antigen*)	(33, 43)	83mM of N-Acetylgalactosamine^*^

* Provided by Vector Laboratories, Inc (Burlingame, CA, USA).

† Provided by GLYcoDiag (Orléans, France).

‡ Provided by Sigma-Aldrich (St. Louis, MI, USA).

Ser, Serine; Thr, Threonine; Fuc, Fucose; GlcNAc, N-acetylglucosamine; GalNAc, N-acetylgalactosamine; Gal, Galactose; Neu5Ac, N-acetylneuraminic Acid; Man, Mannose; Glc, Glucose. mM, millomolar.

The competition was evaluated by a decrease in the mean fluorescence intensity (MFI) signal using the following formula where ‘MFI monosaccharide’ represents staining with pre-incubated lectin binding, ‘cell autofluorescence’ represents the fluorescence without any lectins and ‘MFI’ represents staining with only lectin binding.


$$100-(100x\;\frac{MFI\;monosaccharide-cell\;autofluorescence\;}{MFI-cell\;autofluorescence})$$

The inhibitory test for the sialic acid-binding lectins (MAL-II and SNA) was based on the treatment of the cells with neuraminidase from *Clostridium perfringens* (0.05 U/mL; Sigma-Aldrich, St. Louis, MI, USA) at 37°C for 30 min. After extensive washes with PBS, the treatment efficiency was evaluated using the same formula.

### 2.5 Analysis of membrane glycosylation by flow cytometry

Next, 1 × 10^6^ isolated TMCs or PBMCs were stained with 10 µg/mL of either biotinylated lectins at 4°C for 15 min (**Table 2**). The cells were washed twice and incubated with antibodies defined to identify the different B cell subsets (**Table 3**) and 5 µg/mL streptavidin-FITC (Biolegend, San Diego, CA, USA) at 4°C for 15 min. Thereafter, the cells were washed with PBS at 4°C and analysed using a Cytoflex S flow cytometer (Beckman Coulter, Brea, CA, USA).

**Table 3:** Antibodies used in the flow cytometry analysis.

Antibodies	Clone	Fluorochrome	Manufacturer
Anti-CD5	BL1a	ECD	Beckman Coulter, Brea, CA, USA
Anti-CD19	J3-119	APC Alexa Fluor 700		
Anti-CD24	ALB9	APC Alexa Fluor 750		
Anti-CD24	1A4CD27	PC7		
Anti-CD38	LS198-4-3	PC5.5		
Anti-IgM	SA-DA4	Pacific Blue 450		
Anti-IgD	IA6-2	APC		Biolegend, San Diego, CA, USA

CD, Cluster of differentiation; IgM, immunoglobulin M; IgD, immunoglobulin D; ECD, electron coupled dye; APC, allophycocyanin; PC, phycoerythrin cyanin.

The flow cytometer was standardised using Flow-Set Pro Fluorosphere beads (Beckman Coulter, Brea, CA, USA) to ensure an inter-experimental comparison of the fluorescent intensity over time. Data were analysed using Kaluza 2.1 software (Beckman Coulter, Brea, CA, USA). The B cells were defined as CD19+ after doublet exclusion.

The B cell subsets for the tonsillar samples were determined based on CD38 and IgD staining (44). Bm1 cells were identified as IgD+CD38−, Bm2 cells as IgD+CD38+, Bm2’ cells as IgD+CD38++, Bm3–4 cells as CD19+CD38+IgD−, eBm5 cells as IgD−CD38+, Bm5 cells as IgD−CD38− and plasmablasts (PBs) as CD38+++IgD−.

Peripheral B cell subsets were determined based on CD24, CD38, CD27 and IgD staining. Transitional (TR) B cells were identified as CD24+++CD38+++, naïve (NA) B cells as CD27−IgD+, unswitched memory (UM) B cells as CD27+IgD+, switched memory (SM) B cells as 7CD27+IgD−, PB as CD27+CD38+++ and double negative (DN) B cells as CD27−IgD−.

### 2.6 Analysis of serum glycosylation

#### 2.6.1 Determination of protein concentration in serum

To ensure that any differences in serum glycosylation could not be attributed to variations in the protein amount, the protein concentration in all samples was determined using the bicinchoninic acid assay test (Micro BCA Protein Assay kit, Thermo Scientific, Waltham, MA, USA) in accordance with the manufacturer’s instructions. The serum volume used in the glycosylation tests was then adjusted to contain 7 µg of proteins.

#### 2.6.2 Determination of the glycoprofile of serum proteins using: An ELISA-derived approach

The adjusted serum volume was diluted in PBS and coated in MaxiSorp 96-well Nunc plates under stirring at RT for 2 h. Wells were washed five times in PBS containing 0.05% of Tween 20 (Sigma-Aldrich, St. Louis, MI, USA). Subsequently, 100 µL of biotinylated lectins at an optimised concentration (**Table 4**) were added inside wells under stirring at RT for 1 h. After washing five times, 0.16 µg/mL of horseradish peroxidase (HRP)-conjugated streptavidin (Biolegend, San Diego, CA, USA) was added for 30 min at RT under stirring. After five washes, a coloured reaction was created using tetramethylbenzidine (TMB; Biolegend, San Diego, CA, USA) and stopped with an 11% sulfuric acid solution. The optical density (OD) was determined at 450 nm with a Multiskan GO microplate spectrophotometer and SkanIt software (Thermo Scientific, Waltham, MA, USA).

**Table 4:** Lectins, concentrations and saturating conditions used for ELISA.

Lectins	Concentration (µg/mL)	IgA1 saturating solution	IgA2 saturating solution	IgG saturating solution
AAL	2	oxidized CFBS	oxidized CFBS	oxidized CFBS
ABA	10	oxidized CFBS	oxidized CFBS	oxidized CFBS
ConA	0.2	oxidized CFBS	oxidized CFBS	oxidized 2%BSA
GNA	20	2%BSA	2%BSA	CFBS
GSL-II	10	2%BSA	2%BSA	CFBS
MAL-II	0.5	2%BSA	2%BSA	oxidized CFBS
PHA-L	5	2%BSA	2%BSA	CFBS
PNA	20	2%BSA	2%BSA	oxidized CFBS
RSL	0.5	oxidized CFBS	oxidized 2%BSA	oxidized CFBS
SNA	1	oxidized 2%BSA	2%BSA	oxidized CFBS
UEA	40	2%BSA	2%BSA	CFBS
VVL	40	2%BSA	2%BSA	oxidized CFBS

AAL, Aleuria aurantia lectin; ABA, Agaricus bisporus agglutinin; ConA, Canavalia ensiformis agglutinin; GNA, Galanthus nivalis agglutinin; GSL-II, Griffonia (Bandeiraea) simplicifolia lectin II; MAL-II, Maackia amurensis lectin II; PHA-L, Phaseolus vulgaris leucoagglutinin; PNA, Arachis (Peanut) hypogaea agglutinin; RSL, Ralstonia solanacearum lectin; SNA, Sambucs nigra agglutinin; UEA, Ulex europaeus agglutinin and VVL, Vicia villosa lectin. PBS, Phosphate buffer saline; CFBS, Carbohydrate Free Blocking Solution; BSA, Bovine serum albumin; IgA, Immunoglobulin A; IgG, Immunoglobulin G.

#### 2.6.3 Determination of IgA1, IgA2 and IgG glycosylation by ELISA-derived technique

Further, 0.5 µg/mL of mouse anti-human IgA1 (RM124 clone), mouse anti-human IgA2 (RM125 clone) or mouse anti-human IgG (4A10 clone; Thermo Scientific, Waltham, MA, USA) was coated on MaxiSorp 96-well Nunc plates at 4°C overnight. The plates were washed with a PBS solution containing 0.05% Tween 20 and saturated with either a 2% BSA solution (Sigma-Aldrich, St. Louis, MI, USA) or a carbohydrate-free blocking solution (CFBS; Vector Laboratories, Inc, Burlingame, CA, USA) at 37°C for 1 h depending on the lectin (**Table 4**). An oxidation step was then performed with 0.05 M sodium periodate in 0.05 M pH4 citrate buffer (Sigma-Aldrich, St. Louis, MI, USA) to cleave terminal glycans present on the anti-Ig and saturating buffer. Next, 100 µL of 1:100 diluted sera was added and incubated at 37°C for 1 h. After five washes, biotinylated lectins (**Table 4**) were added to the plate under stirring at RT for 1 h. After five washes, 0.16 µg/mL of HRP-conjugated streptavidin was added under stirring at RT for 30 min. After five additional washes, a TMB solution was added, and the coloured reaction was stopped with 11% sulfuric acid. The OD was determined at 450 nm using a Multiskan GO microplate spectrophotometer and SkanIt software (Thermo Scientific, Waltham, MA, USA).

### 2.7 Statistical analysis

GraphPad Prism 9 software was used to plot graphs and conduct a statistical analysis. The normal distribution was verified, and then two groups were compared using the Mann–Whitney U test, while more than two groups were compared using a one-way ANOVA test and Dunn’s multiple comparison test without adjustment. The correlation between age and glycosylation was determined by linear regression with *p*-value< 0.05 and R2 > 0.50.

## 3 Results

**Figure 2:**
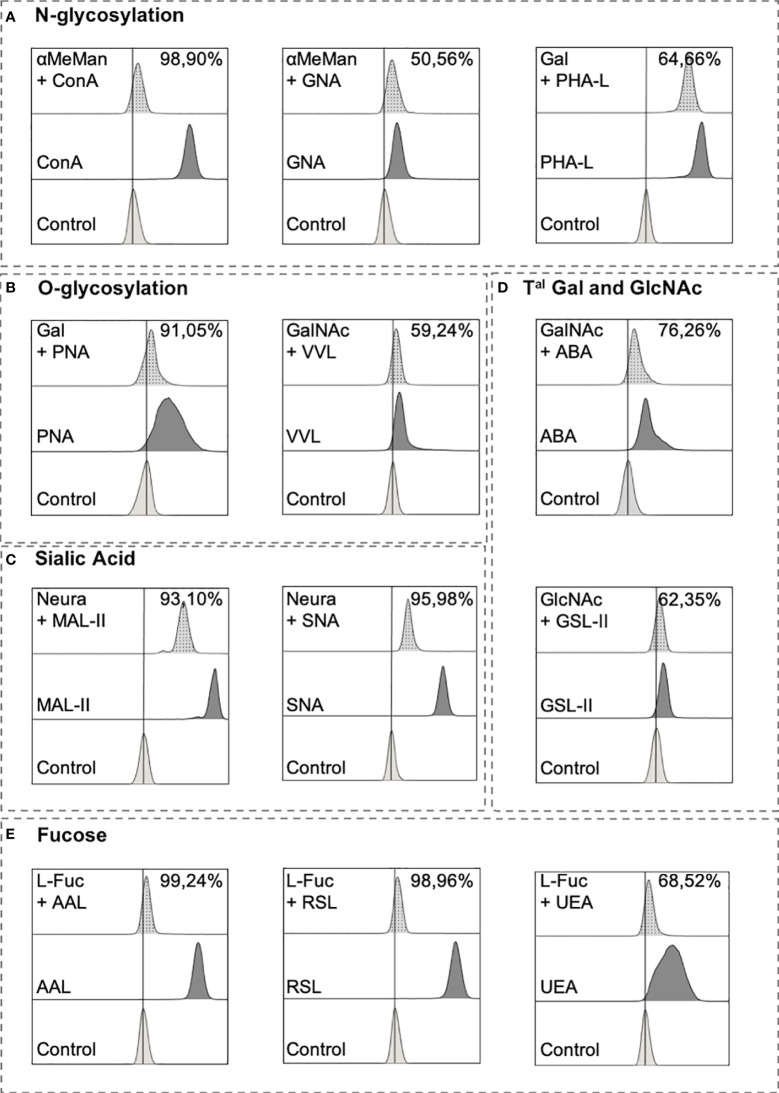
Lectin inhibitory tests.Lectins (except MAL-II and SNA) (**Table 2**) were pre-incubated with a PBS solution containing monosaccharides for 15 min at 4°C with PBS before being used to stain Ramos cell line for 15 min at 4°C. After 2 washes in PBS, cells were incubated for 15 min at 4°C with 5 µg/mL streptavidin-FITC. After wash with PBS, cells were analyzed using Beckman Coulter Cytoflex S. For ConA and GNA, inhibition test consists in pre-incubating lectins with α-methyl-mannoside (360mM). PHA-L and PNA were pre-incubated with 200 mM of galactose (Gal). N-acetylgalactosamine (GalNAc) was pre-incubated at 83mM with VVL and ABA. GSL-II was pre-incubated with 200 mM of N-acetylglucosamine (GlcNAc) and 200mM of L-fucose (L-Fuc) was used for AAL, RSL and UEA lectins. For MAL-II and SNA, cells were treated with neuraminidase (0.05 U/mL) and washed twice followed with a regular staining procedure. Results are presented as followed: N-glycosylation in **(A)**, O-glycosylation in **(B)**, Sialylation in **(C)**, Terminal (T^al^) Gal and GlcNAc on N and O-glycosylation in **(D)**, and Fucosylation in **(E)**.

We evaluated the lectin specificity to validate the use of flow cytometry to stain with lectins. Several monosaccharide agonists were incubated with lectins before performing the staining (**Table 2**). As shown in **Figure 2**, the fluorescent intensity of the signal obtained with the mannose-binding ConA and GNA decreased by 98.90% and 50.56%, respectively, when lectins were pre-incubated with α-methyl-mannoside (aMeMan; 360 mM). When Gal was used at 200 mM, PHA-L and PNA binding decreased by 64.66% and 91.05%. For lectins that recognise GalNAc, 83mM of GalNAc was sufficient to decrease the ABA and VVL bindings by 76.26% and 59.24%, respectively. For the fucose-binding lectins, 200 mM of L-fucose (L-Fuc) was sufficient to decrease AAL, RSL and UEA bindings by 99.24%, 98.96% and 68.52%, respectively. Thereafter, the cells were pre-treated with neuraminidase (0.05 U/mL) for 30 min at 37°C for lectins that recognise sialic acid (MAL-II and SNA), and the fluorescent signal obtained with SNA and MAL-II decreased by 95.27% and by 93.10%, respectively. These results were expected for the lectins that had low inhibition percentages (GNA, PHA-L, VVL, ABA, GSL-II and UEA) because they recognise motifs included in a series of glycans and because we used free monosaccharides. Although these conditions do not exactly reflect the reality of complex glycans, they allowed us to validate the specificity of these tools for further study.

### 3.1 Association of tonsillar B cell maturation process with important N-glycan and O-glycan remodelling

A panel of antibodies was designed to identify any change in the glycosylation status of the different B cell subsets (**Table 3**). Antibodies can modify the cell glycoprofile because they carry glycans. Thus, biotinylated lectins were added first, followed by B cell markers. We used this established experimental protocol to perform the lectin staining procedure on the tonsillar cells. Functional B cell subsets can be distinguished using several membrane proteins. In particular, variations in the expression levels of IgD and CD38 have been used to develop a model for mature B cell homeostasis through germinal centre (GC) cells (44). This model suggests that naive Bm1 cells (IgD+CD38−) become Bm2 cells (IgD+CD38+) once they are activated and then develop into GC founder Bm2′ cells (IgD+CD38++). These cells can differentiate into centroblast Bm3 cells, centrocyte Bm4 cells (IgD−CD38++) and eventually early memory Bm5 cells (IgD–CD38+) and memory Bm5 cells (IgD−CD38−) or plasma cells (PB). **Figure S1A** depicts the gating strategy for assessing these different tonsillar CD19+ B cell subsets.

**Figure 3:**
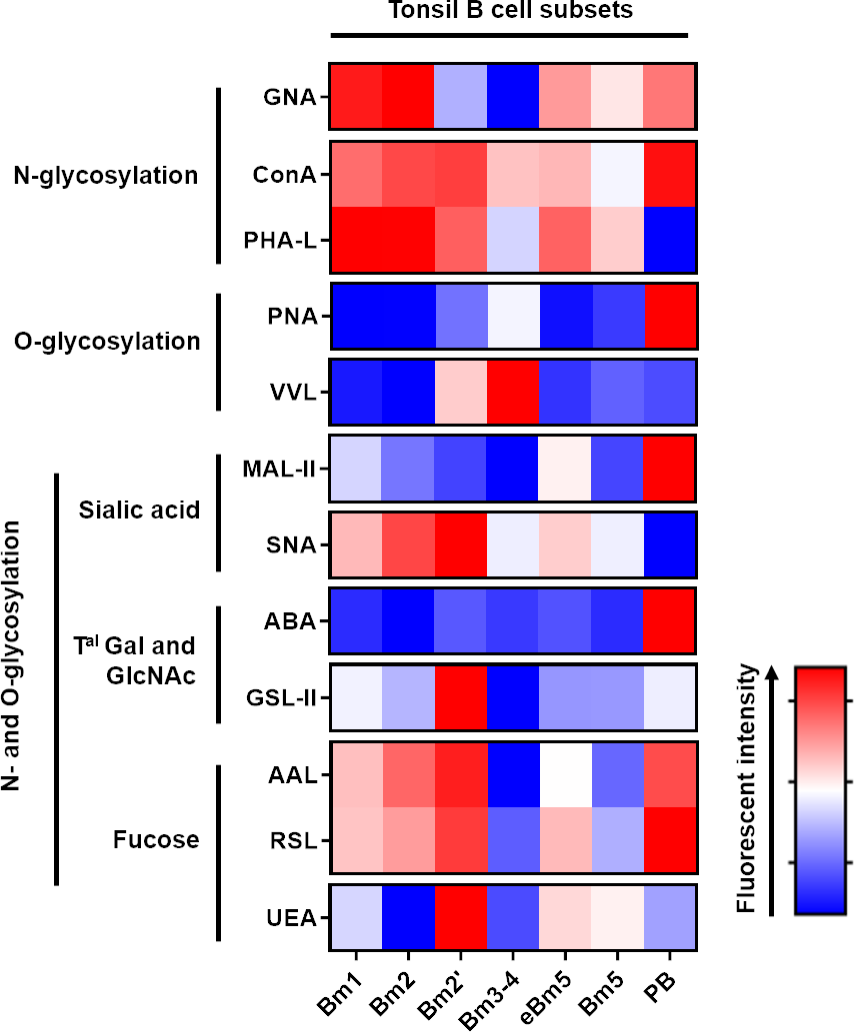
Tonsillar B cell maturation process is associated with an important N- and O-glycan remodeling. Freshly isolated tonsillar mononuclear cells were stained with lectins and the specific B cell antibody panel before analysis using Beckman Coulter Cytoflex S. Fluorescence intensity is expressed as geomean. Freshly isolated tonsillar mononuclear cells were stained with lectins (**Table 2**).

We were able to identify a differential presence of sugar patterns for most of the lectins, depending on the maturation status of the B cells (**Figure 3**). Although no statistical difference was obtained with ConA, a significant decrease in the presence of terminal mannose on N-glycans was mainly observed in the Bm3–4 B cells using GNA, and the transition towards eBm5, Bm5 and PB was accompanied by a restoration of an expression level nearly similar to those of Bm1 and Bm2. In terms of β1-6 branched N-glycans, Bm3–4 and PB were characterised by a reduction in the staining obtained with PHA-L when compared to other states (**Figure S2A**). In the case of T and Tn antigens, which were identified using PNA and VVL lectins, respectively, a marked expression in the GC B cells (Bm2’ and Bm3–4) was observed as expected. Significantly high staining with PNA was identified on PB, indicating the presence of additional T antigens (**Figure S2B**). When MAL II was used, a progressive decrease in α2-3 sialylation was observed from the Bm1 to Bm3–4 cells. The transition towards eBm5 and Bm5 was accompanied by a complete restoration. Interestingly, the final maturation in PB was associated with an important increase in α2-3 sialic acids. SNA did not reveal any difference in the presence of α2-6 sialic acids on the different B cell subsets (**Figure S2C**). The same was observed with ABA and GSL II (Gal-GlcNAc/Gal-GalNAc and terminal GlcNAc, respectively; **Figure S2D**). In terms of fucosylation, AAL staining (α1-3/4/6 fucose linked to GlcNAc residues) mainly decreased on the Bm3–4 cells, while RSL and UEA staining, which targeted α1-6 fucose linked to GlcNAc and α1-2 fucose linked to Gal residues, respectively, did not exhibit any statistical difference in the different B cell subsets (**Figure S2E**).

Altogether, our flow cytometry analysis using lectins revealed that tonsillar B cell maturation is associated with profound changes in glycans at the cell surface.

### 3.2 Association of peripheral B cell differentiation with a profound remodelling of N- and O- glycans at the cell surface similar to that in tonsils

**Figure 4:**
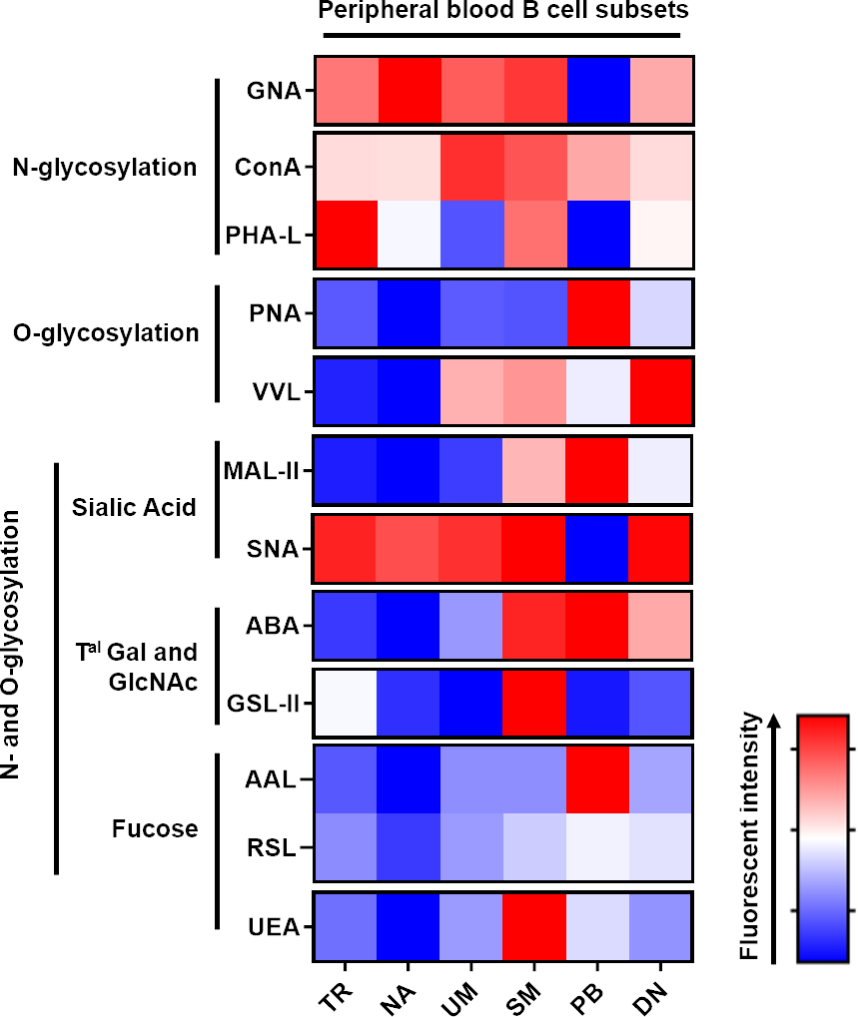
Peripheral blood B cell differentiation is associated with a rearrangement of N and O-glycan surface and an increase of Galactose α2-3 N-acetylneuraminic acid residues. Freshly isolated peripheral blood mononuclear cells (PBMC) were stained with lectins the specific B cell antibody panel before analysis using Beckman Coulter Cytoflex S. Fluorescence intensity is expressed as geomean. All the listed annotation stands for; TR: Transitional B cells; NA: Naive B cells; UM: Unswitched-memory B cells; SM: Switched-memory B cells; PB: Plasmablasts and DN: Double negative (CD27^-^IgD^-^) B cells; Gal: Galactose; Tal: terminal; GlcNAc: N-acetylglucosamine. Freshly isolated tonsillar mononuclear cells were stained with lectins (**Table 2**) and the specific B cell antibody panel.

A cohort of 30 HDs was selected to analyse the blood glycome of B cells and their subsets (**Table 2**). The most immature peripheral B cell population in humans has been characterised in detail by the concomitantly high expression of CD24 and CD38 (45). B cells have been divided into four distinct populations using the surface expression of IgD and CD27 as a surrogate marker for human memory B cells. IgD+CD27− B cells represent the NA B cell pool, whereas the expression of CD27 and loss of surface IgD expression on B cells are features of classical SM B cells. B cells that express CD27 and IgD have been characterised as UM B cells. The delineation of human memory B cells by CD27 expression has been challenged by the characterisation of CD27-negative B cells (IgD−CD27−), also called DN, indicating molecular imprints of memory B cells (somatic hypermutation and immunoglobulin class-switch) (46). The gating strategy is shown in **Figure S1B**. First, the effects of gender and age on the glycoprofile of each B cell subset were evaluated. As presented in **Figure S3A**, no statistical difference was observed between the male and female HDs. We also assessed if age could affect the glycoprofiles. As shown in **Figures S3B** (age of men) and S3C (age of women), no direct correlation between age and the glycan signature could be established. We were able to identify a differential presence of sugar patterns for most of the lectins, depending on the diffetentiation state of the B cells (**Figure 4**).

Although no statistical difference was obtained with ConA, the presence of terminal mannose on N-glycans (GNA) was remarkably decreased on PB (**Figure S4A**). Considering the results obtained with PHA-L, an important loss in β1-6 branched N glycans was assessed in UM and PB (**Figure S4A**). The DN B cells and PB were characterised by an increase in the amount of T and Tn antigens (targeted with PNA and VVL lectins, respectively; **Figure S4B**). Compared to the SM, PB and DN B cells, the NA B cells exhibited a decreased level of α2-3 sialic acids, which were targeted by MAL-II (**Figure S4C**). A discrete decrease in PB was the only difference that was observed for α2-6, which was recognised by SNA. When stained with ABA, NA exhibited a significant decrease in staining when compared to the SM and PB subsets, and the detection of terminal GlcNAc motive by GSL-II was similar across the subsets with a discrete increase in SM. (**Figure S4D**). Regarding the fucosylation of B cell surface markers, there was no major difference between each subset with the three lectins used (AAL, RSL and UEA), except for a slight decrease in NA B cells, an increase in SM and DN compared to NA and a discrete increase in PB (**Figure S4E**).

Analysis of the glycosylation of B cell subsets from peripheral blood demonstrated significant remodelling of membrane glycans similar to that of tonsils in accordance with the different steps of the differentiation process.

### 3.3 B cells and their subsets from pSS and SLE patients are carrying altered glycoprofiles

**Figure 5:**
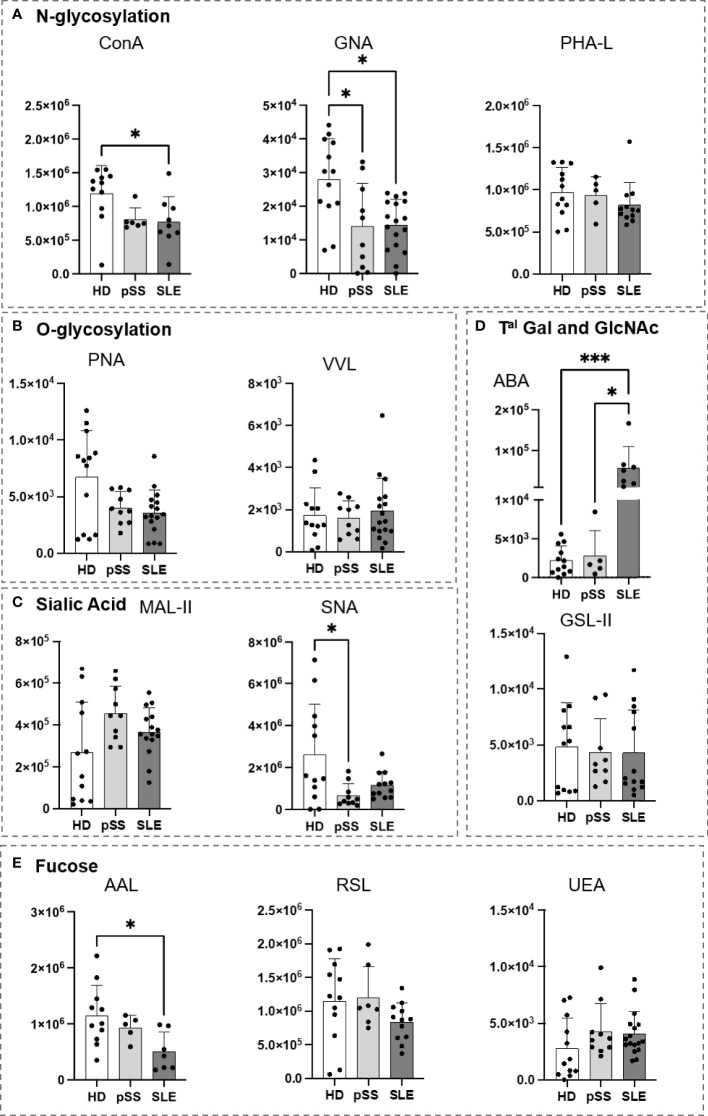
Analysis of B cell glycosylation reveals deep alterations in primary Sjögren’s syndrome (pSS) and systemic lupus erythematosus (SLE). Freshly isolated peripheral blood mononuclear cells (PBMC) (10^6^) from 13 healthy donors (HD), 10 pSS, and 17 SLE patients were stained with lectins at 10 µg/mL for 15 min at 4°C (**Table 2**). After two wash steps, cells were incubated with streptavidin-FITC (5 µg/mL) and an antibody panel (**Table 3**) for 15 min at 4°C. After staining and washing in PBS at 4°C, cells were analyzed using Beckman Coulter Cytoflex S. Fluorescence intensity from CD19+ B cells is expressed as geomean. Results are presented as followed: N-glycosylation in **(A)**, O-glycosylation in **(B)**, Sialylation in **(C)**, Terminal (T^al^) Gal and GlcNAc on N and O-glycosylation in **(D)**, Fucosylation in **(E)**. One-way anova test followed by Dunn’s multiple comparison test were performed. ns: not significant p > 0,05, stars are indicated when p< 0,05 (*); < 0,001 (***).

As presented in **Figure 5**, the B cell glycoprofiles of pSS and SLE patients were compared to those of HDs. Regarding N-glycosylation, the SLE B cells demonstrated a decrease in Con A staining when compared to the HD B cells (**Figure 5A**). Compared to the HDs, the pSS and SLE patients showed a significant reduction in terminal or high-mannose (GNA) in their B cells. O-glycosylation (PNA and VVL) exhibited no difference (**Figure 5B**). Regarding the sialic acids, although staining with MAL-II exhibited no difference, the staining with SNA revealed desialylated B cells in the pSS patients. A similar observation was made with SLE B cells without reaching any statistical significance (**Figure 5C**). When ABA was used, the SLE B cells exhibited a stronger signal than the HD and pSS B cells (**Figure 5D**). Regarding fucosylation, RSL and UEA exhibited no differences, while AAL revealed that the SLE B cells presented a loss in α1-3/4/6 fucose linked to GlcNAc residues (**Figure 5E**).

The glycoprofile of the different B cell subsets (TR, NA, UM, SM, DN and PB) of these two AIDs was assessed, and the results are presented in **Supplementary Figure 5**. A summary of the differences is presented in **Table 5**.

**Table 5:** Schematic representation of the glycoprofile obtained for each B cell subsets presented in the **Supplementary Figure 4**.

	TR	NA	UM	SM	PB	DN	TR	NA	UM	SM	PB	DN	TR	NA	UM	SM	PB	DN		
GNA																			N-glycans	
ConA																				
PHA-L																				
PNA																			O-glycans	
VVL																				
MAL-II																			Sialic acid	N and O-glycosylation
SNA																				
ABA																			T^al^ Gal and GlcNAc	
GSL-II																				
AAL																			Fucose	
RSL																				
UEA																				
	**HD vs pSS**						**HD vs SLE**						**pSS vs SLE**							

TR, Transitional B cells; NA, Naïve B cells; UM, Unswitched memory B cells; SM, Switched memory B cells; PB, Plasmablasts; DN, Double negative memory B cells.

The white square represents no statistical difference between the two compared groups.

The blue square represents a significant decrease in AID vs HD.

The red square represents a significant increase in AID vs HD.

The purple square represents a significant increase in SLE vs pSS.

The NA and UM cells in the pSS patients presented less high-mannose structures (GNA, ConA) than those in the HDs. The SM B cells carried few β1-6 branched N-glycan structures and T antigen on their surface markers. These discrepancies were associated with an increased level of α2-3 sialic acids (recognised by MAL II) and a decrease in α2-6 (SNA). There were no changes in the glycoprofiles of TR, PB and DN.

Compared to those in the HDs, all the B cell subsets in the SLE patients carried reduced levels of high-mannose structures (Con A and GNA), except for PB, which is consistent with our previous observation on total B cells. The SM B cells presented few β1-6 N-glycan motifs. A significant afucosylation (staining with AAL) was observed on the NA, UM, SM and DN B cells. The SLE DN B cells also displayed few high-mannose motives and significant afucosylation. The most remarkable result was obtained with ABA, which indicated that all the SLE B cell subsets carried more terminal Gal-GlcNAc/Gal-GalNAc elements when compared to the B cells from the HDs and pSS patients. We also noticed that SLE PB had more Tn antigens than pSS PB.

Compared to the HD B cells, the pSS and SLE B cells and their subsets presented important changes in their glycoprofiles. All these modifications could be responsible for the physiological activity and reactivity in those patients.

### 3.4 Serum proteins from SLE patients are carrying more Gal-GlcNAc/Gal-GalNAc motives

**Figure 6:**
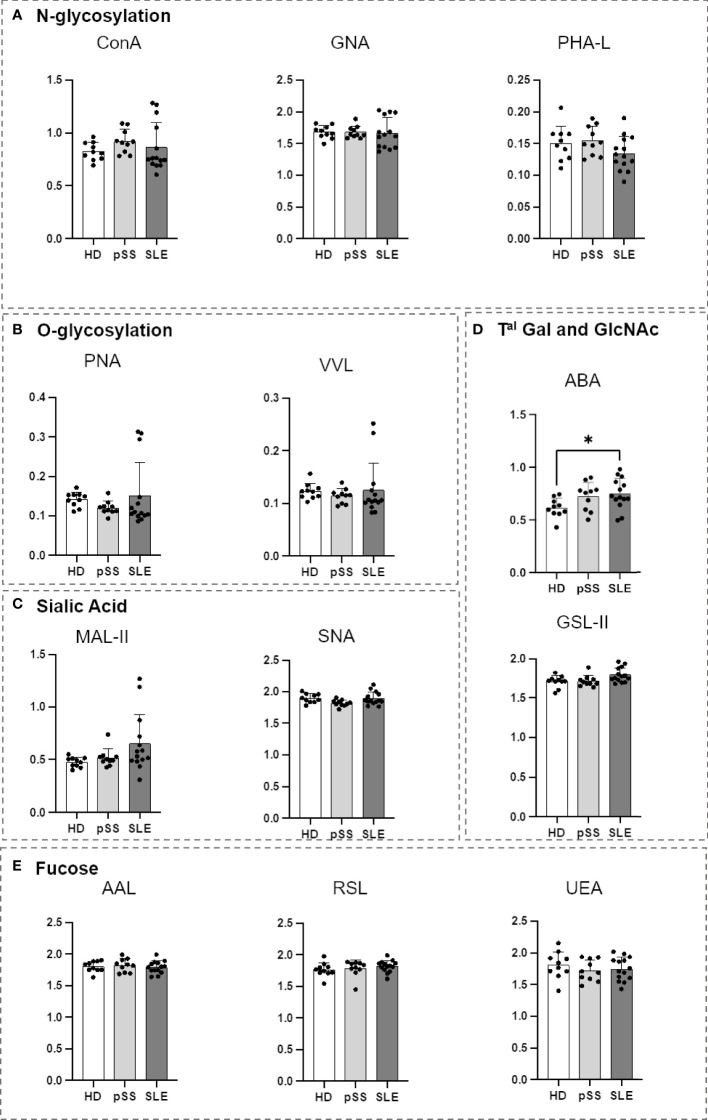
Comparison of serum protein N- and O-glycosylation from systemic lupus erythematosus (SLE), primary Sjögren’s syndrome (pSS) and healthy donors (HD). First, the serum protein concentration from healthy donors (HD), primary Sjögren’s syndrome (pSS) and systemic lupus erythematosus (SLE) patients (10 for each) was quantified using the bicinchoninic acid assay test. The equivalent of 7 µg of serum protein was used from each patient, diluted in PBS and coated in MaxiSorp 96-well Nunc plates for 2 h at room temperature (RT). After 5 washes with PBS supplemented with 0,05% Tween 20, biotinylated lectins (**Table 2**) were added at optimized concentration for 1 h at RT. After 5 wash steps streptavidin-Horse Radish Peroxydase (HRP) was added for 30 min at RT. After 5 wash steps, Tetramethylbenzidine (TMB) was added was added for 8 min and the reaction was stopped with sulfuric acid. The optical density (OD) at 450 nm was determined for each lectin. Results are presented as followed: N-glycosylation in **(A)**, O-glycosylation in **(B)**, Sialylation in **(C)**, Terminal (T^al^) Gal and GlcNAc on N and O-glycosylation in **(D)**, Fucosylation in **(E)**. One-way anova test followed by Dunn’s multiple comparison test were performed. ns, not significant p > 0,05, stars are indicated when p< 0,05 (*).

An ELISA-derived approach was developed using biotinylated lectins as a revealing agent to study the glycosylation of serum proteins. Sera from the pSS and SLE patients were compared to those from the HDs (**Table 1**). Only ABA demonstrated a significant difference, indicating that the serum proteins from the SLE patients carried more Gal-GlcNAc/Gal-GalNAc motives (**Figure 6**). This result supports the results obtained for the SLE B cells and their subsets. This suggests that there were some impairments in this same glycosylation pathway.

### 3.5 IgA1 and IgA2 from the pSS and SLE patients carried less high-mannose structures and more GlcNAc residues, whereas IgG had less α2-6 sialic acid and hypofucosylation

**Figure 7:**
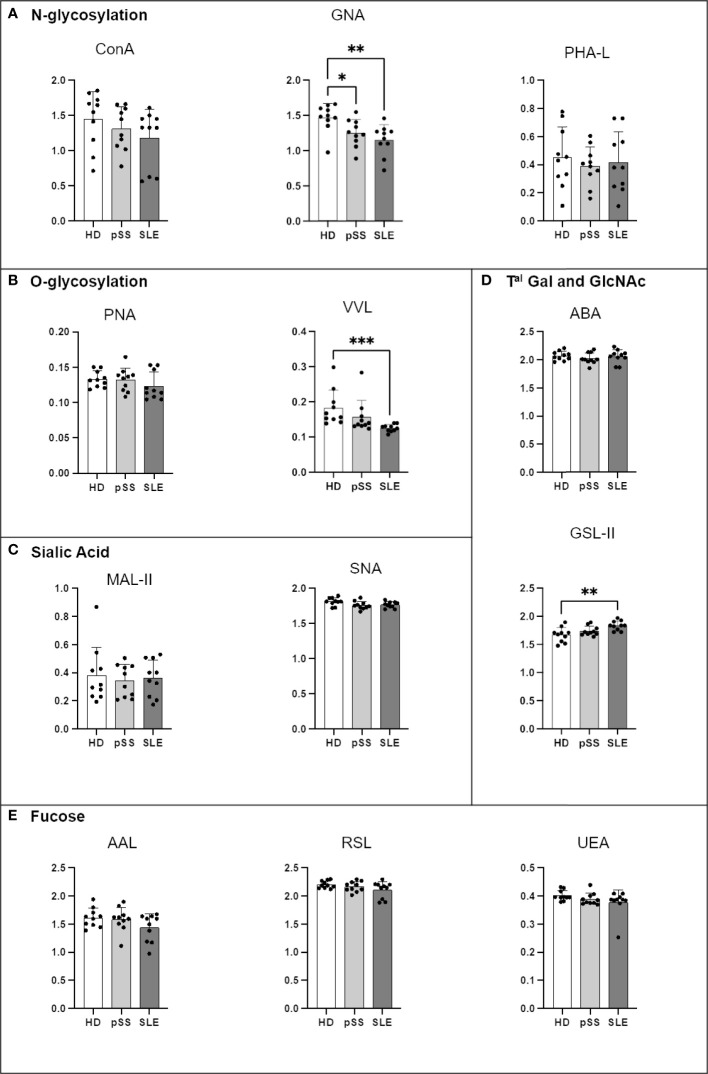
Comparison of IgA1 N- and O-glycosylation from systemic lupus erythematosus (SLE), primary Sjögren’s syndrome (pSS) and healthy donors (HD). Mouse anti-Human IgA1 (RM124 clone) was coated at 0.5 µg/mL in MaxiSorp 96-well plates overnight at 4°C. A PBS solution containing 2% of Bovine Serum Albumin (BSA) or Carbohydrate-Free Blocking Solution were used for saturation depending on the lectins. This information is described in **Table 4**. After saturation, an oxidation was performed, to cleave terminal glycans present on anti-Ig and saturating buffer, with a solution of sodium periodate at the final concentration of 0,0 5M in 0,05 M pH=4 citrate buffer. Serum from healthy donors (HD), primary Sjögren’s syndrome (pSS) and SLE diluted to 1/100e were added for 1 h at 37°C. After 5 wash steps with PBS 0,05% Tween20, the biotinylated lectins described in **Table 2** were added for 1 h. Plate was washed 5 times and streptavidin-Horse Radish Peroxydase (HRP) was added for 30 min at for 2 h at room temperature (RT). After 5 wash steps, a colored reaction using tetramethylbenzidine was performed and stopped with sulfuric acid. The optical density (OD) at 450 nm was determined. Results are presented as followed: N-glycosylation in **(A)**, O-glycosylation in **(B)**, Sialylation in **(C)**, Terminal (T^al^) Gal and GlcNAc on N and O-glycosylation in **(D)**, Fucosylation in **(E)**. One-way anova test followed by Dunn’s multiple comparison test were performed. ns, not significant p > 0,05, stars are indicated when p< 0,05 (*) < 0,01 (**); < 0,001 (***).

Another ELISA-derived assay was optimised to focus on IgA1, IgA2 and IgG in order to further our investigations. Compared to that from the HDs, IgA1 from the pSS and SLE patients carried fewer terminal mannose residues on N-glycans revealed by GNA. IgA1 from the SLE patients also presented less VVL staining (Tn antigen) and more terminal GlcNAc (GSL-II lectin) motives than that from the HDs, indicating a decrease in terminal galactosylation. No other difference was observed (**Figure 7**).

**Figure 8:**
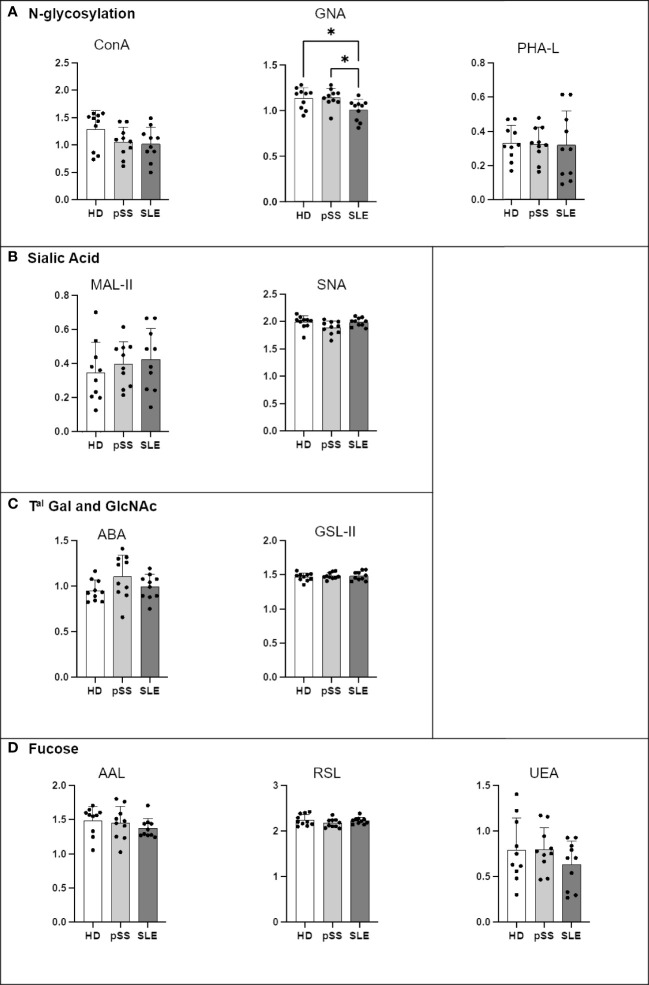
Comparison of IgA2 N-glycosylation from systemic lupus erythematosus (SLE), primary Sjögren’s syndrome (pSS) and healthy donors (HD). Mouse anti-Human IgA2 (RM125 clone) was coated at 0.5 µg/mL in MaxiSorp 96-well plates overnight at 4°C. A PBS solution containing 2% of BSA or Carbohydrate-Free Blocking Solution were used for saturation depending on the lectins. This information is described in **Table 4**. After saturation, an oxidation was performed, to cleave terminal glycans present on anti-Ig and saturating buffer, with a solution of sodium periodate at the final concentration of 0,05 M in 0,05 M pH=4 citrate buffer. Serum from healthy donors (HD), pSS and SLE diluted to 1/100e were added for 1 h at 37°C. After 5 wash steps with PBS 0,05% Tween20, the biotinylated lectins described in **Table 2** were added for 1 h. Plate was washed 5 times and streptavidin-HRP was added for 30 min at RT. After 5 wash steps, a colored reaction using tetramethylbenzidine was performed and stopped with sulfuric acid. The optical density (OD) at 450 nm was determined. Results are presented as followed: N-glycosylation in **(A)**, Sialylation in **(B)**, Terminal (Tal) Gal and GlcNAc on N and O-glycosylation in **(C)**, and Fucosylation in **(D)**. One-way anova test followed by Dunn’s multiple comparison test were performed. ns: not significant p > 0,05, stars are indicated when p< 0,05 (*).

IgA2 from the SLE patients presented less terminal mannose on N-glycans (GNA) than that from the HDs and pSS patients (**Figure 8**). No other significant difference was observed. The ELISA for PNA and VVL was not conducted because IgA2 had no O-glycosylation site.

**Figure 9:**
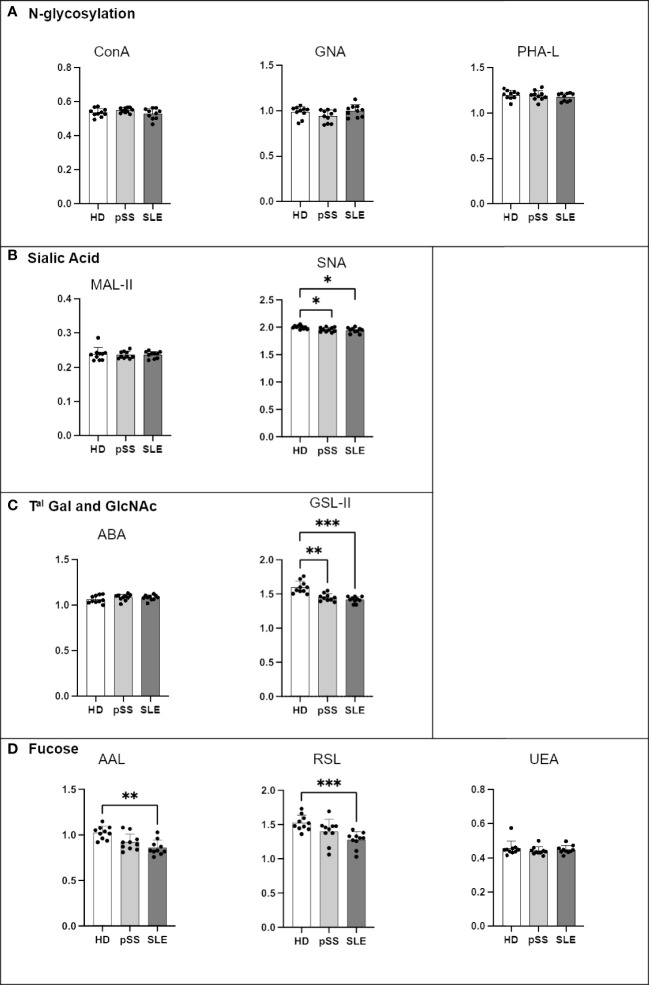
Comparison of IgG N-glycosylation from systemic lupus erythematosus (SLE), primary Sjögren’s syndrome (pSS) and healthy donors (HD). Mouse anti-Human IgG (4A10 clone) was coated at 0.5 µg/mL in MaxiSorp 96-well plates overnight at 4°C. A PBS solution containing 2% of BSA or Carbohydrate-Free Blocking Solution were used for saturation depending on the lectins. This information is described in **Table 4**. After saturation, an oxidation was performed, to cleave terminal glycans present on anti-Ig and saturating buffer, with a solution of sodium periodate at the final concentration of 0,05 M in 0,0 5M pH=4 citrate buffer. Serum from healthy donors HD, pSS and SLE diluted to 1/100e were added for 1h at 37°C. After 5 wash steps with PBS 0,0 5% Tween20, the biotinylated lectins described in **Table 2** were added for 1h. Plate was washed 5 times and streptavidin-HRP was added for 30 min at RT. After 5 wash steps, a colored reaction using tetramethylbenzidine was performed and stopped with sulfuric acid. The optical density (OD) at 450 nm was determined. Results are presented as followed: N-glycosylation in **(A)**, Sialylation in **(B)**, Terminal (Tal) Gal and GlcNAc on N and O-glycosylation in **(C)**, and Fucosylation in **(D)**. One-way anova test followed by Dunn’s multiple comparison test were performed. ns: not significant p > 0,05, stars are indicated when p< 0,05 (*); < 0,01 (**); < 0,001 (***).

The analysis of the IgG glycoprofiles from the AID patients revealed that the N-glycan residues (ConA, GNA and PHA-L) exhibited no difference (**Figure 9**). A significant decrease in α2-6 sialylation was observed on pSS and SLE IgG (SNA staining) along with a low terminal GlcNAc binding attested by GSL-II. Finally, compared to that from the HDs, IgG from the SLE patients presented a decrease in α1-3/4/6 fucose linked to GlcNAc residues (AAL and RSL) but not in α1-2 fucose linked to Gal (UEA). PNA and VVL were not tested because IgG had no O-glycosylation site.

## 4 Discussion

Glycosylation plays a key role in physiology and protein secretion. It is also involved in cell proliferation, apoptosis and differentiation (47). Surface glycans change their structure and complexity during the maturation and differentiation of B lymphocytes. We observed this phenomenon in our tonsillar and peripheral blood B cells. In summary, normal B cell differentiation and maturation are accompanied by a decrease in N-glycan complexity, sialic acid and fucose but an increase in T and Tn O-glycans. Specifically, two types of cells are mostly impacted by these modifications: Bm3/4 in tonsils and PB in tonsils and PBMCs. Giovannone et al. (2018) revealed that Bm3/4 in tonsils (GC B cells) express more T and Tn antigens to acquire specific functions such as antigen receptor signalling (48, 49). In the case of PB, the production of antibodies is accompanied by a decrease in complex N-glycans, an increase in T antigens and an increase in α2-3 sialic acid expression (48, 49). In the case of PB, the production of antibodies is accompanied by a decrease in complex N-glycans, an increase in T antigens and an increase in α2-3 sialic acid expression (48). Antigen receptors are essential for B cells, and they are post-translationally modified with N-glycan and O-glycan chains during the maturation process of these cells (50). These modifications change the receptor trafficking, interactions with their cell surface markers, and interactions with the glycoproteins from other cell types. They can also alter the transduced signals from the BCR to the cells directly or indirectly by perturbing the activity of inhibitory proteins such as CD22 (51, 52). All these findings demonstrate the importance of glycosylation in controlling B cell behaviour.

**Figure 10:**
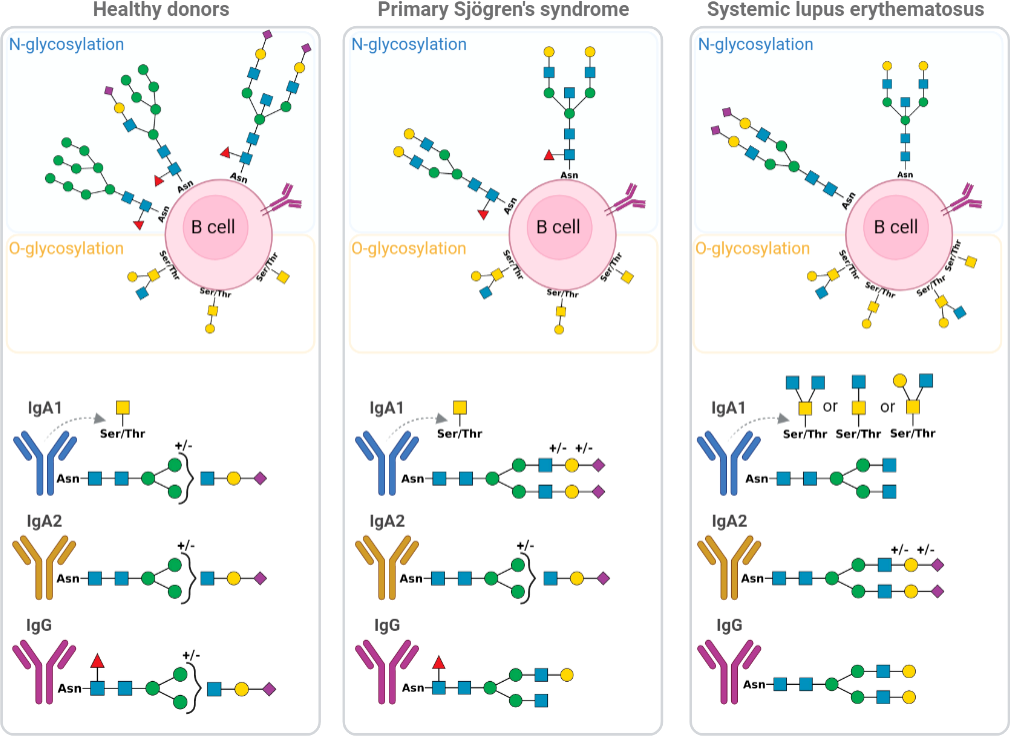
Schematic representation of glycosylation alteration of B cell and immunoglobulins IgA1, IgA2 and IgG from primary Sjögren’s syndrome (pSS) patients and systemic lupus erythematosus (SLE) patients compared to healthy donors (HD). The different forms of N and O-glycosylation on B cell surface are presented as well as IgA1, IgA2 and IgG glycan. Green circle: Mannose; yellow circle: Galactose; yellow square: N-acetylgalactosamine; blue square: N-acetylglucosamine; purple diamond; Sialic acid and red triangle; Fucose. Asn, Asparagine; Thr, Threonine; Ser, Serine.

Deciphering any changes in the global glycoprofile of B cells and their subsets in SLE and pSS diseases could aid in understanding the loss of self-tolerance and the physiopathology of these two AIDs. As described in **Figure 10**, B cells from AIDs undergo abnormal glycosylation. Both pSS and SLE exhibit increased N-glycan complexity during the early stages of differentiation. Interestingly, α2-6 sialic acid is absent in pSS B cells and barely present in SLE, whereas α-fucose is reduced only in SLE B cells. In-depth characterisations have revealed that memory B cells are remarkably altered, and these glycosylation changes could explain the positive selection of autoreactive B cells (49, 53), and the increased activation of BCR caused by the absence of CD22 sialic acid ligands (39, 52, 54). In contrast to pSS B cells, SLE B cells exhibit Gal-GlcNAc/Gal-GalNAc terminal patterns, suggesting a lack of terminal glycosylation. All these phenomena could affect BCR signalling and antibody production by B cells in AIDs (21, 55).

Indeed, antibody production is affected by B cells that are altered by glycosylation; immunoglobulins could also be affected. As demonstrated, only IgA1 from SLE had large amounts of core 2 O-glycans, whereas IgA2 had small amounts of Tal mannose. Additionally, both the pSS and SLE patients exhibited hyposialylated IgG with terminal Gal, whereas only the SLE patient exhibited a decrease in core fucose. These alterations could affect the fixation of IgG to the FcyRIIIa receptor and induce the antibody-dependent cell-mediated cytotoxicity (ADCC) phenomenon (56) and C1q recruitment for complement-dependent cytotoxicity (CDC) reactions (57, 58), thereby promoting tissue inflammation. All these results are presented in **Figure 10**.

It is worth noting that serum proteins, including immunoglobulins, can exhibit aberrant fucosylation and sialylation, which are exclusively found on B cell surface markers. No similar observation was made on T cells from these same patients (data not shown). This finding indicates that there are profound alterations in the biology and functions of B cells in AIDs. These modifications could be due to modified gene expression or mutation, certain epigenetic modifications and transfection factors present at the wrong moment during the B cell differentiation process. As demonstrated by Cao et al., the presence of cytokine in the microenvironment of B cells could regulate intracellular glycosyltransferase and lead to changes in Ig glycans patterns (59). Wang and coworkers observed that plasma cell membrane glycosylation and IgG1 secreted immunoglobulins glycosylation is affected depending on the activation stimuli of naive B cells (60).

All these modification may be the result of modulated expression of glycosylation-related genes or the modulation of enzymes activity. All these perturbations result in the presence of different immune cells (such as B cells) with a perturbed behaviour. For example, they may carry some receptors with excessive or inefficient affinities for their ligands and/or secrete structurally modified proteins such as antibodies, causing different perturbations in clearance and reactivity to (self)-antigens. All these biological steps can amplify these perturbations mildly or severely, off-setting the immune system homeostasis towards some aggressive inflammation processes. Glycobiology appears to be a more complex science, and significant inquiries at all these levels (glycosylation-enzyme expression and activity for example) are required to understand and manage it. Nevertheless, our simple experimental approaches, such as serum protein glycoprofile analysis, could be used as a biomarker to develop new therapeutic strategies.

## Data availability statement

The original contributions presented in the study are included in the article/**Supplementary Material**. Further inquiries can be directed to the corresponding author.

## Ethics statement

The studies involving human participants were reviewed and approved by “CRB Santé de Brest” (BB-0033-00037) and “Centre de référence des maladies auto-immunes rares” Brest, France. Written informed consent to participate in this study was provided by the participants’ legal guardian/next of kin.

## Author contributions

MM, PP, AB, CB and MD designed the study. MM, PP and AB wrote the first draft of the manuscript and figures. MM, PP and WE performed and analyzed the experiments. VD-P, SJ-J and DC gave access to patient’s cohorts. CJ and J-OP participated in the text edition. All authors contributed to the article and approved the submitted version.

## Funding

The research leading to these results has received support from the Innovative Medicines Initiative Joint Undertaking under the Grant Agreement Number 115565 (PRECISESADS project), resources of which are composed of financial contribution from the European Union’s Seventh Framework Program (FP7/2007–2013) and EFPIA companies’ in-kind contribution.

## Acknowledgments

The authors would like to acknowledge CRB “Centre de Ressources Biologiques” and CERAINO « Centre de référence des maladies auto-immunes rares », Brest, France, for collection of blood samples from patients, the Hyperion flow core facilities, for their technical help. We thank also Dr Eléonore Betacchioli and Dr Baptiste Chevet for diagnostic cohort’s information. We also thank Brest Metropole Oceane for supporting this project.

## Conflict of interest

The authors declare that the research was conducted in the absence of any commercial or financial relationships that could be construed as a potential conflict of interest.

## Publisher’s note

All claims expressed in this article are solely those of the authors and do not necessarily represent those of their affiliated organizations, or those of the publisher, the editors and the reviewers. Any product that may be evaluated in this article, or claim that may be made by its manufacturer, is not guaranteed or endorsed by the publisher.

## Supplementary material

Supplementary Figure 1 Tonsillar mononuclear cells (TMC) and peripheral blood mononuclear cells (PBMC) gating strategies. **(A)** After the doublet removal, within TMC, B cells were identified as expressing CD19: subpopulations were defined as followed based on expression of CD38 and IgD: Bm1 as CD38^-^IgD^+^, Bm2 as CD38^+^IgD^+^, Bm2’ as CD38^++^IgD^+^, Bm3-4 cells as CD19^+^CD38^+^IgD^-^, eBm5 as CD38^+^IgD^-^, Bm5 as CD38^-^IgD^-^ and PB as CD38^+++^IgD^-^. One representative experiment (out of 20) is shown. **(B)** After the doublet removal, within PBMC, B cells were identified as expressing CD19: subpopulations were defined as followed: Plasmablast (PB) as CD38^hi^IgD^-^, Transitional (TR) B cells as CD24^hi^CD38^hi^. After excluding TR B cells, we defined double negative (DN) B cells as CD27^-^IgD^-^, naïve (NA) B cells as CD27^-^IgD^+^, unswitched memory (UM) B cells as CD27^+^IgD^+^ and switched memory (SM) B cells as CD27^+^IgD^-^. One representative experiment (out of 30) is shown.

Supplementary Figure 2 Tonsillar B cell maturation process is associated with an important N- and O-glycan remodeling. Freshly isolated tonsillar mononuclear cells were stained with lectins and the specific B cell antibody panel before analysis using Beckman Coulter Cytoflex S. Fluorescence intensity is expressed as geomean. Results are presented as followed: N-glycosylation in **(A)**, O-glycosylation in **(B)**, Sialylation in **(C)**, Terminal (T^al^) Gal and GlcNAc on N and O-glycosylation in **(D)**, and Fucosylation in **(E)**. One-way anova test followed by Dunn’s multiple comparison test were performed. ns, not significant p > 0,05, stars are indicated when p< 0,05 (*);< 0,01 (**);< 0,001 (***);<0,0001 (****).

Supplementary Figure 3 Sexe and Age parameters do not influence B cell glycosylation. Freshly isolated peripheral blood mononuclear cells (PBMC) (10^6^) from 30 healthy donors (HD) including 18 Male (M) and 12 Female (F) were stained with lectins at 10 µg/mL for 15 min at 4°C (). After two wash steps, cells were incubated with streptavidin-FITC (5 µg/mL) and an antibody panel () for 15 min at 4°C. After staining and washing in PBS at 4°C, cells were analyzed using Beckman Coulter Cytoflex S. Fluorescence intensity is expressed as geomean. Results are presented as followed: N-glycosylation in **(A)**, O-glycosylation in **(B)**, Sialylation in **(C)**, Terminal (T^al^) Gal and GlcNAc on N and O-glycosylation in **(D)**, Fucosylation in **(E)**. **(3A)**. Comparison of B cell glycosylation between Male and Female from healthy donor group. Mann-Whitney T test was performed. ns: not significant p > 0,05, stars are indicated when p< 0,05 (*);< 0,01 (**);< 0,001 (***);<0,0001 (****). **(3B)**. Correlation between Male age and B cell glycosylation. Linear regression was performed. ns, not significant p > 0,05 and R^2^ > 0.5. **(3C)**. Correlation between Female age and B cell glycosylation. Linear regression was performed. ns, not significant p > 0,05 and R^2^ > 0.5.

Supplementary Figure 4 Peripheral blood B cell differentiation is associated with a rearrangement of N and O-glycan surface and an increase of Galactose α2-3 N-acetylneuraminic acid residues. Freshly isolated peripheral blood mononuclear cells (PBMC) were stained with lectins the specific B cell antibody panel before analysis using Beckman Coulter Cytoflex S. Fluorescence intensity is expressed as geomean. Results are presented as followed: N-glycosylation in **(A)**, O-glycosylation in **(B)**, Sialylation in **(C)**, Terminal (T^al^) Gal and GlcNAc on N and O-glycosylation in **(D)**, and Fucosylation in **(E)**. One-way anova test followed by Dunn’s multiple comparison test were performed. ns, not significant p > 0,05, stars are indicated when p< 0,05 (*);< 0,01 (**);< 0,001 (***);<0,0001 (****). All the listed annotation stands for; TR, Transitional B cells; NA, Naive B cells; UM, Unswitched-memory B cells; SM, Switched-memory B cells; PB, Plasmablasts and DN, Double negative (CD27^-^IgD^-^) B cells.

Supplementary Figure 5 The analysis of B cell subset glycosylation reveals deep alterations in primary Sjögren’s syndrome (pSS) and systemic lupus erythematosus (SLE) compared to healthy donors. Freshly isolated peripheral blood mononuclear cells (PBMC) (10^6^) from 13 healthy donors (HD), 10 pSS, and 17 SLE patients were stained with lectins at 10 µg/mL for 15 min at 4°C (). After two wash steps, cells were incubated with streptavidin-FITC (5 µg/mL) and an antibody panel () for 15 min at 4°C. After staining and washing in PBS at 4°C, cells were analyzed using Beckman Coulter Cytoflex S. Fluorescence intensity is expressed as geomean. HD, pSS and SLE glycosylation of transitional B cell **(5A)**, naïve B cells **(5B)**, unswitched-memory B cells **(5C)**, switched-memory B cells **(5D)**, plasmablasts **(5E)** and double negative CD27^-^IgD^-^ B cells **(5F)** was analyzed. Results are presented as followed: N-glycosylation in A, O-glycosylation in B, Sialylation in C, Terminal (T^al^) Gal and GlcNAc and terminal GlcNAc on N and O-glycosylation in D, Fucosylation in E. One-way anova test followed by Dunn’s multiple comparison test were performed. ns, not significant p > 0,05, stars are indicated when p< 0,05 (*);< 0,01 (**);< 0,001 (***);<0,0001 (****).
