# Supplementary figures and images for: Abnormal B cell glycosylation in autoimmunity: A new potential treatment strategy
Source: Front Immunol. 2022 Aug 25;13:975963. doi: 10.3389/fimmu.2022.975963 (PMC9453492; doi:10.3389/fimmu.2022.975963)

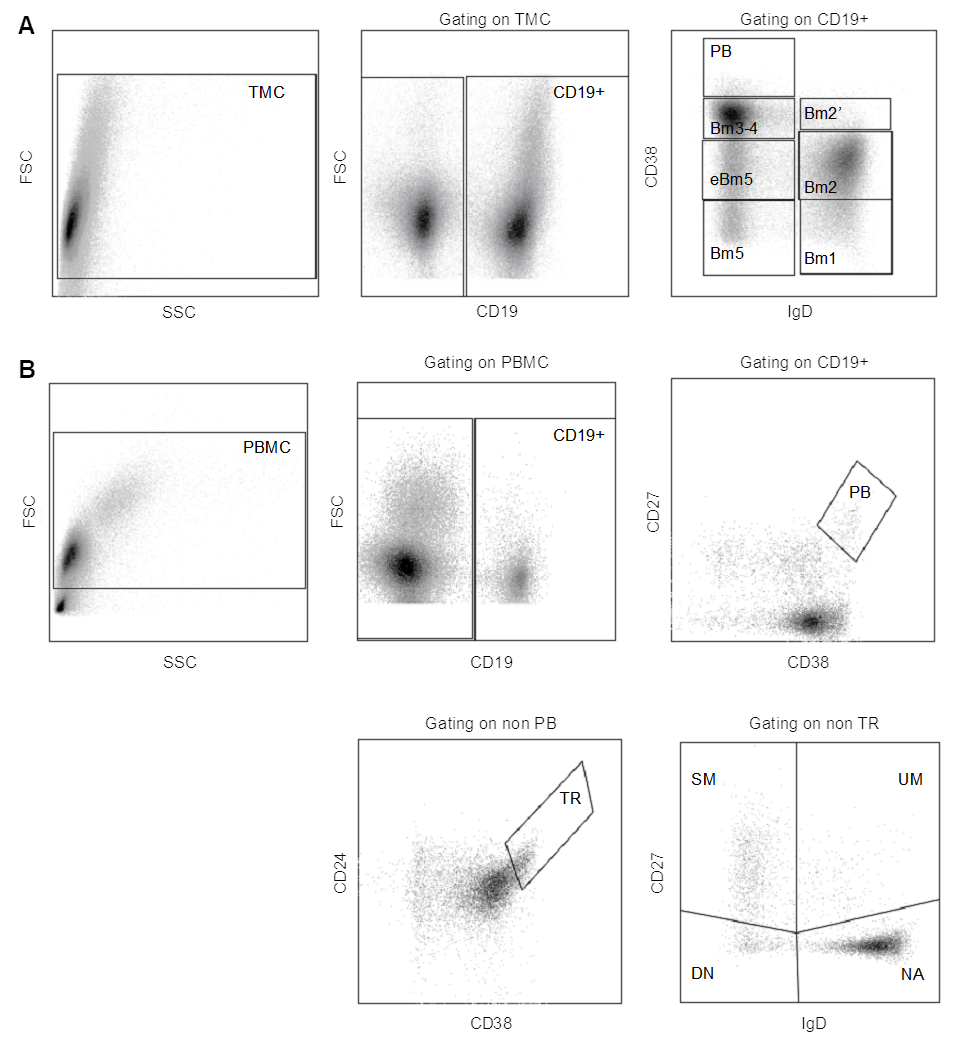

Supplement: Supplementary Figure 1 — Tonsillar mononuclear cells (TMC) and peripheral blood mononuclear cells (PBMC) gating strategies. (A) After the doublet removal, within TMC, B cells were identified as expressing CD19: subpopulations were defined as followed based on expression of CD38 and IgD: Bm1 as CD38-IgD+, Bm2 as CD38+IgD+, Bm2’ as CD38++IgD+, Bm3-4 cells as CD19+CD38+IgD-, eBm5 as CD38+IgD-, Bm5 as CD38-IgD- and PB as CD38+++IgD-. One representative experiment (out of 20) is shown. (B) After the doublet removal, within PBMC, B cells were identified as expressing CD19: subpopulations were defined as followed: Plasmablast (PB) as CD38hiIgD-, Transitional (TR) B cells as CD24hiCD38hi. After excluding TR B cells, we defined double negative (DN) B cells as CD27-IgD-, naïve (NA) B cells as CD27-IgD+, unswitched memory (UM) B cells as CD27+IgD+ and switched memory (SM) B cells as CD27+IgD-. One representative experiment (out of 30) is shown. [file Image_1.tif]

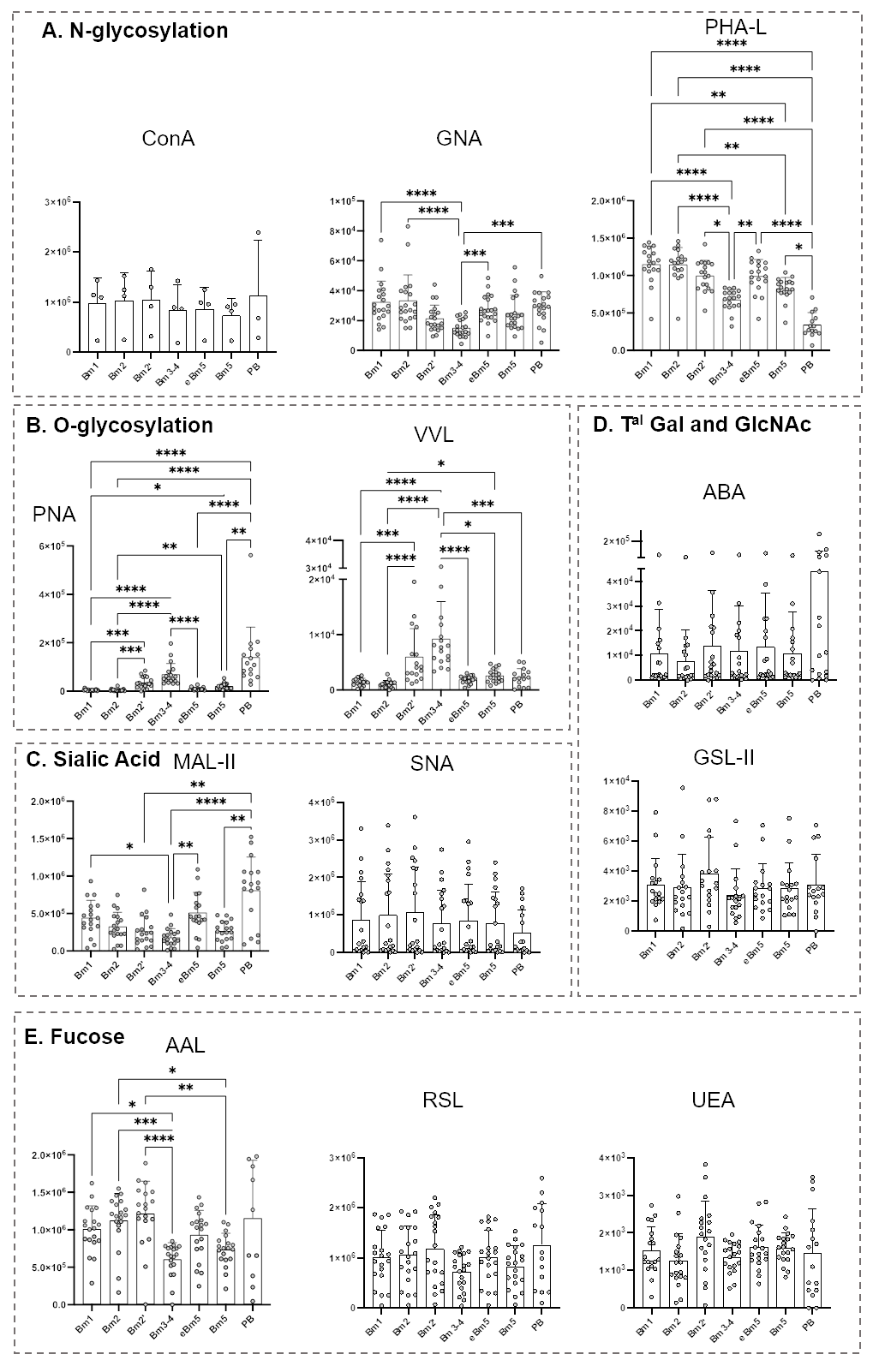

Supplement: Supplementary Figure 2 — Tonsillar B cell maturation process is associated with an important N- and O-glycan remodeling. Freshly isolated tonsillar mononuclear cells were stained with lectins and the specific B cell antibody panel before analysis using Beckman Coulter Cytoflex S. Fluorescence intensity is expressed as geomean. Results are presented as followed: N-glycosylation in (A), O-glycosylation in (B), Sialylation in (C), Terminal (Tal) Gal and GlcNAc on N and O-glycosylation in (D), and Fucosylation in (E). One-way anova test followed by Dunn’s multiple comparison test were performed. ns, not significant p > 0,05, stars are indicated when p< 0,05 (*);< 0,01 (**);< 0,001 (***);<0,0001 (****). [file Image_2.tif]

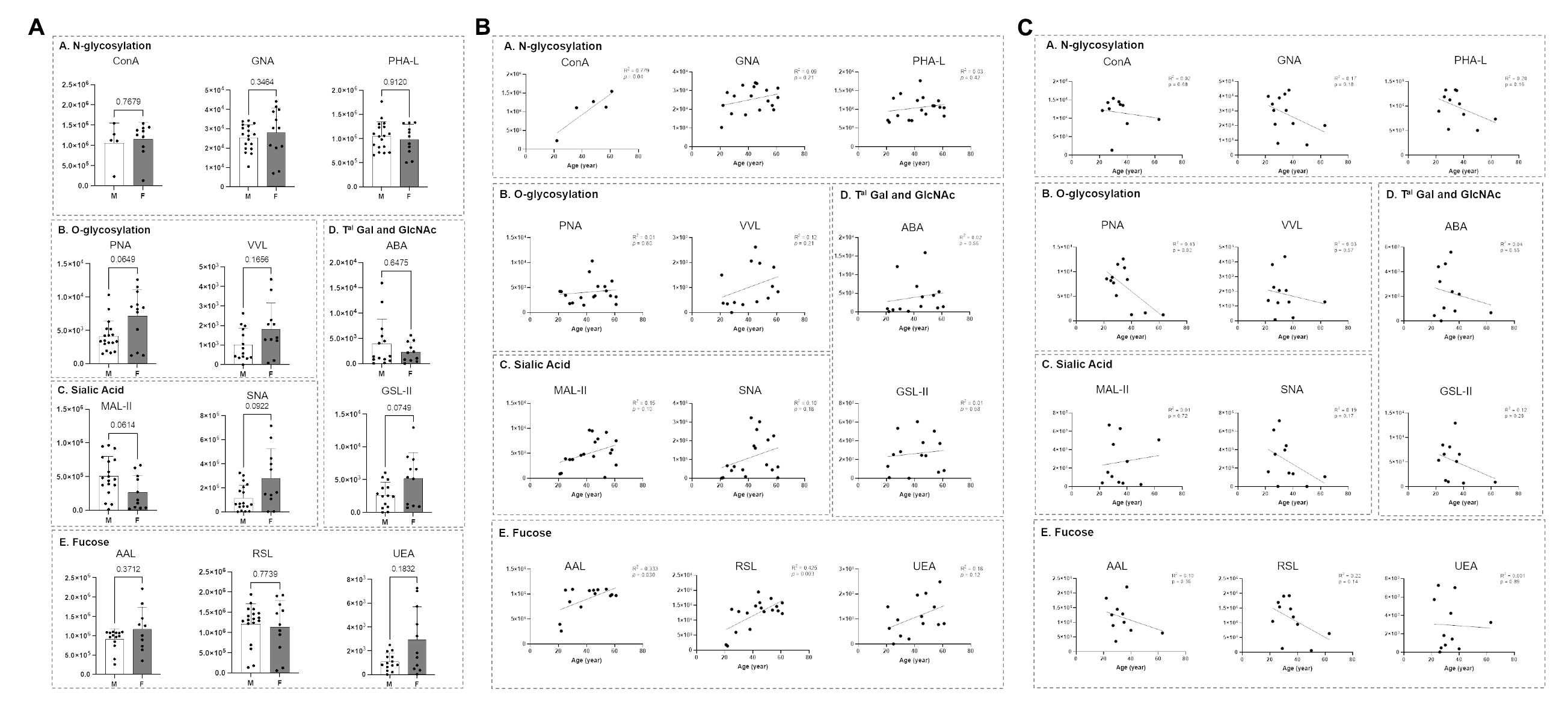

Supplement: Supplementary Figure 3 — Sexe and Age parameters do not influence B cell glycosylation. Freshly isolated peripheral blood mononuclear cells (PBMC) (106) from 30 healthy donors (HD) including 18 Male (M) and 12 Female (F) were stained with lectins at 10 µg/mL for 15 min at 4°C (). After two wash steps, cells were incubated with streptavidin-FITC (5 µg/mL) and an antibody panel () for 15 min at 4°C. After staining and washing in PBS at 4°C, cells were analyzed using Beckman Coulter Cytoflex S. Fluorescence intensity is expressed as geomean. Results are presented as followed: N-glycosylation in (A), O-glycosylation in (B), Sialylation in (C), Terminal (Tal) Gal and GlcNAc on N and O-glycosylation in (D), Fucosylation in (E). (3A). Comparison of B cell glycosylation between Male and Female from healthy donor group. Mann-Whitney T test was performed. ns: not significant p > 0,05, stars are indicated when p< 0,05 (*);< 0,01 (**);< 0,001 (***);<0,0001 (****). (3B). Correlation between Male age and B cell glycosylation. Linear regression was performed. ns, not significant p > 0,05 and R2 > 0.5. (3C). Correlation between Female age and B cell glycosylation. Linear regression was performed. ns, not significant p > 0,05 and R2 > 0.5. [file Image_3.tif]

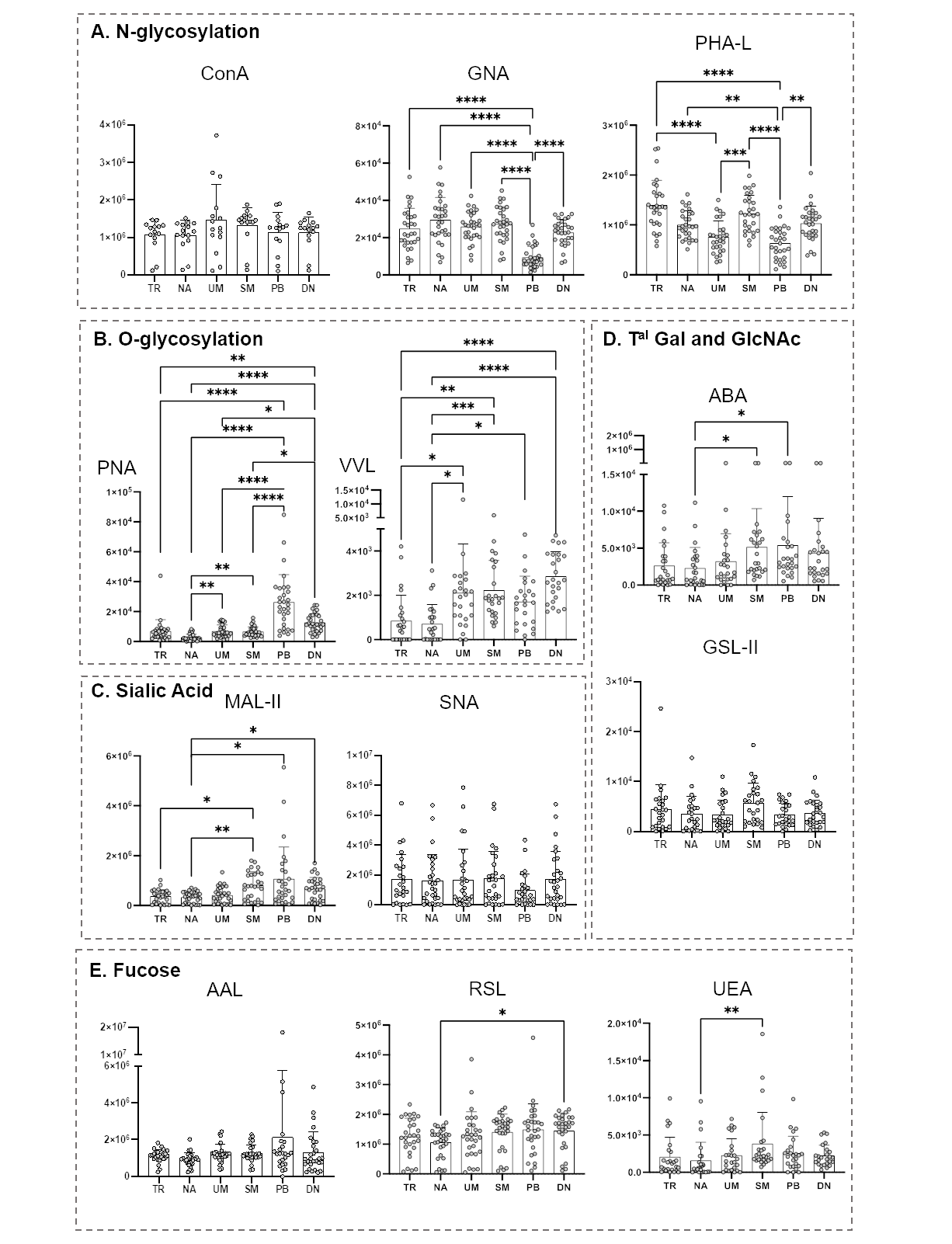

Supplement: Supplementary Figure 4 — Peripheral blood B cell differentiation is associated with a rearrangement of N and O-glycan surface and an increase of Galactose α2-3 N-acetylneuraminic acid residues. Freshly isolated peripheral blood mononuclear cells (PBMC) were stained with lectins the specific B cell antibody panel before analysis using Beckman Coulter Cytoflex S. Fluorescence intensity is expressed as geomean. Results are presented as followed: N-glycosylation in (A), O-glycosylation in (B), Sialylation in (C), Terminal (Tal) Gal and GlcNAc on N and O-glycosylation in (D), and Fucosylation in (E). One-way anova test followed by Dunn’s multiple comparison test were performed. ns, not significant p > 0,05, stars are indicated when p< 0,05 (*);< 0,01 (**);< 0,001 (***);<0,0001 (****). All the listed annotation stands for; TR, Transitional B cells; NA, Naive B cells; UM, Unswitched-memory B cells; SM, Switched-memory B cells; PB, Plasmablasts and DN, Double negative (CD27-IgD-) B cells. [file Image_4.tif]

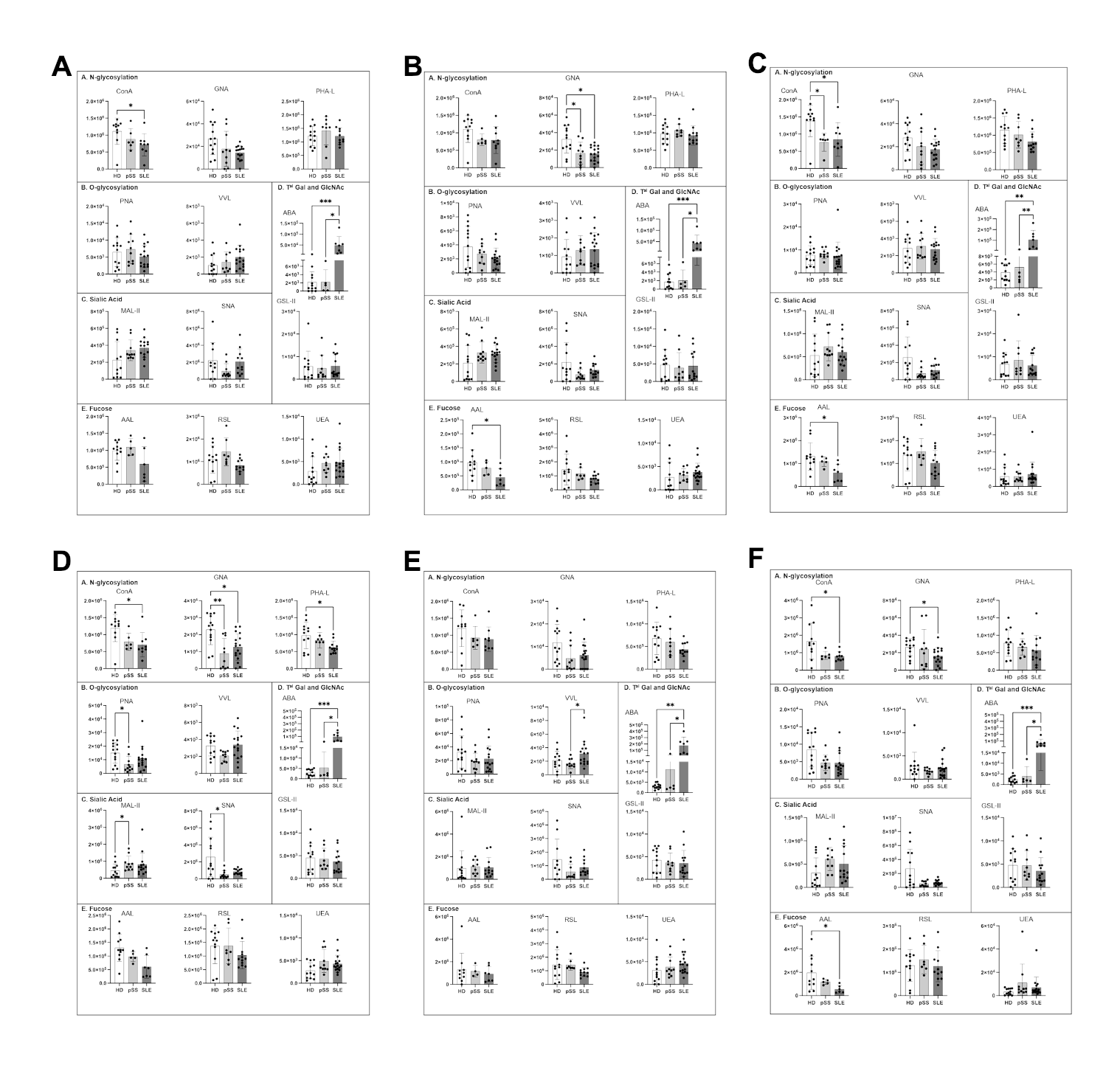

Supplement: Supplementary Figure 5 — The analysis of B cell subset glycosylation reveals deep alterations in primary Sjögren’s syndrome (pSS) and systemic lupus erythematosus (SLE) compared to healthy donors. Freshly isolated peripheral blood mononuclear cells (PBMC) (106) from 13 healthy donors (HD), 10 pSS, and 17 SLE patients were stained with lectins at 10 µg/mL for 15 min at 4°C (). After two wash steps, cells were incubated with streptavidin-FITC (5 µg/mL) and an antibody panel () for 15 min at 4°C. After staining and washing in PBS at 4°C, cells were analyzed using Beckman Coulter Cytoflex S. Fluorescence intensity is expressed as geomean. HD, pSS and SLE glycosylation of transitional B cell (5A), naïve B cells (5B), unswitched-memory B cells (5C), switched-memory B cells (5D), plasmablasts (5E) and double negative CD27-IgD- B cells (5F) was analyzed. Results are presented as followed: N-glycosylation in A, O-glycosylation in B, Sialylation in C, Terminal (Tal) Gal and GlcNAc and terminal GlcNAc on N and O-glycosylation in D, Fucosylation in E. One-way anova test followed by Dunn’s multiple comparison test were performed. ns, not significant p > 0,05, stars are indicated when p< 0,05 (*);< 0,01 (**);< 0,001 (***);<0,0001 (****). [file Image_5.tif]
